# Effects of Climate, Plant Height, and Evolutionary Age on Geographical Patterns of Fruit Type

**DOI:** 10.3389/fpls.2021.604272

**Published:** 2021-03-16

**Authors:** Tong Lyu, Yunyun Wang, Ao Luo, Yaoqi Li, Shijia Peng, Hongyu Cai, Hui Zeng, Zhiheng Wang

**Affiliations:** ^1^School of Urban Planning and Design, Shenzhen Graduate School, Peking University, Shenzhen, China; ^2^Institute of Ecology and Key Laboratory of Earth Surface Processes of Ministry of Education, College of Urban and Environmental Sciences, Peking University, Beijing, China; ^3^National Engineering Laboratory for Applied Technology of Forestry & Ecology in Southern China, College of Life Science and Technology, Central South University of Forestry and Technology, Changsha, China

**Keywords:** fleshy fruit, geographic pattern, angiosperm, China, plant height, macroevolution

## Abstract

Fruit type is a key reproductive trait associated with plant evolution and adaptation. However, large-scale geographical patterns in fruit type composition and the mechanisms driving these patterns remain to be established. Contemporary environment, plant functional traits and evolutionary age may all influence fruit type composition, while their relative importance remains unclear. Here, using data on fruit types, plant height and distributions of 28,222 (∼ 90.1%) angiosperm species in China, we analyzed the geographical patterns in the proportion of fleshy-fruited species for all angiosperms, trees, shrubs, and herbaceous species separately, and compared the relative effects of contemporary climate, ecosystem primary productivity, plant height, and evolutionary age on these patterns. We found that the proportion of fleshy-fruited species per grid cell for all species and different growth forms all showed significant latitudinal patterns, being the highest in southeastern China. Mean plant height per grid cell and actual evapotranspiration (AET) representing ecosystem primary productivity were the strongest drivers of geographical variations in the proportion of fleshy-fruited species, but their relative importance varied between growth forms. From herbaceous species to shrubs and trees, the relative effects of mean plant height decreased. Mean genus age had significant yet consistently weaker effects on proportion of fleshy-fruited species than mean plant height and AET, and environmental temperature and precipitation contributed to those of only trees and shrubs. These results suggest that biotic and environmental factors and evolutionary age of floras jointly shape the pattern in proportion of fleshy-fruited species, and improve our understanding of the mechanisms underlying geographical variations in fruit type composition. Our study also demonstrates the need of integrating multiple biotic and abiotic factors to fully understand the drivers of large-scale patterns of plant reproductive traits.

## Introduction

Plants show remarkable diversity in their fruit types, varying from tiny dry fruits to huge fleshy fruits. Fruit type is a key reproductive trait for the evolution of plant diversity (Coombe, 1976; Obeso, 2004). It has been shown that evolutionary changes in fruit type are normally associated with shifts in plant diversification rate (Tiffney, 1984; Eriksson and Bremer, 1992; Beaulieu and Donoghue, 2014). Moreover, fruit type is associated with life-history strategies that are related to competitive ability of species in communities and species adaptation to environmental changes (Traveset et al., 2004; Lotan and Izhaki, 2013), suggesting that fruit type may represent an important factor affecting community assembly and community dynamics in response to climate change. Exploring geographical variations in fruit type composition in plant communities across large spatial scales and their mechanisms will shed light on the mechanisms underlying large-scale patterns in reproductive traits and their responses to climate change. Contemporary climate (Almeida-Neto et al., 2008; Chen et al., 2017; Zhao et al., 2018), growth forms (Bolmgren and Eriksson, 2005), plant functional traits (e.g., plant height) and evolutionary age may all influence geographical variations in fruit type composition, while the relative contribution of different factors remains poorly understood.

Previous studies have explored the relationship between fruit type composition and contemporary climate, especially precipitation and temperature (Traveset et al., 2004; Chen et al., 2017; Zhao et al., 2018). Studies find that fleshy fruits, especially the succulent pericarp, require high water supply from plants when they become ripe (Lotan and Izhaki, 2013; Yu et al., 2017), which suggests that fleshy-fruited species may be more prevalent in regions with high water availability. In addition, the susceptibility of fleshy-fruited species to freezing temperature (Burke et al., 1976) and high frugivore abundance in unseasonal environments (Lin et al., 2009) may lead the prevalence of fleshy-fruited species in regions with warm and unseasonal climate. In general, areas with sufficient water and energy availability tend to have high ecosystem primary productivity, and hence a positive correlation between the prevalence of fleshy-fruited species and ecosystem primary productivity is also expected. Previous studies have indicated that, to support the multiplication of cell numbers and the synthesis of large quantities of compounds (e.g., terpenes and esters) during the maturation of fleshy fruits (Coombe, 1976; Seymour et al., 2013), the production of fleshy fruits may require more energy than the production of dry fruits. We therefore expect that fleshy-fruited species are more prevalent in regions with high ecosystem primary productivity.

In addition to the effect of contemporary climate, the prevalence of fleshy-fruited species has also been found to be associated with plant size (e.g., mature height) and canopy closeness (Butler et al., 2007). In general, communities with taller plants tend to have more closed vegetation due to larger leaves and canopy area (Díaz et al., 2016), and hence more shaded and complex understory environment. Previous studies suggest that in closed vegetation with shaded understory, seed dispersal of plant species heavily relies on animals (Lorts et al., 2008). Fleshy fruits represent one of the adaptations of plants to attract animal dispersers, especially frugivores (e.g., primates and birds) (Wheelwright and Orians, 1982; Moles et al., 2006; Bolmgren and Eriksson, 2010). Therefore, fleshy-fruited species are expected to be more prevalent in communities with taller plants and more closed canopy. Moreover, compared with shorter plants, taller plants often have better access to light and larger leaf area, and hence have better capacity to gain energy for reproductive investment (Cornelissen, 1999; Falster and Westoby, 2003). Therefore, taller plants tend to have better chances to produce fleshy fruits than shorter ones (Leishman and Westoby, 1994; Moles and Westoby, 2004). These findings again suggest that fleshy-fruited species would be more prevalent in communities dominated by taller species. However, the relationship between fruit type composition and plant height in communities remains to be quantified.

Evolutionary history may also lead to variation in fruit type composition across floras of different regions (Lorts et al., 2008). Previous studies suggest that early angiosperms at ca. 130 Ma before the present had small and abiotically dispersed seeds and dry fruits (Crane et al., 1995; Eriksson et al., 2000). Moreover, recent phylogenetic studies also indicate that dry-fruited angiosperm species are more prevalent in basal and old lineages in the current tree of life and in different clades [e.g., Solanaceae in Knapp (2002) and Campanulids in Beaulieu and Donoghue (2014)]. In contrast to the early origination of dry fruit, fleshy fruit may have originated since only ca. 70 Ma ago and more than half of fleshy-fruited clades may have originated during the last 40 Ma (Bolmgren and Eriksson, 2005; Eriksson, 2016). In other words, compared with dry fruit, fleshiness is likely a derived trait (Tiffney, 1984; Mack, 2000) and is much younger than dryness. These findings suggest that fleshy-fruited species are expected to be more prevalent in regions with younger floras than with older ones. However, due to lack of data, the relationship between fruit type composition and evolutionary age of floras across large spatial scales remains unexplored.

Using data of fruit types of all angiosperms in China, together with data of high-resolution species distributions, we demonstrated the geographic pattern in the composition of angiosperm fruit types (particularly the proportions of fleshy-fruited species) in China and evaluated the climatic, biotic, and evolutionary drivers underlying these patterns. We aim to test the following three hypotheses: (1) fleshy-fruited species are more prevalent in areas with higher ecosystem primary productivity; (2) the proportion of fleshy-fruited species increases with plant height; (3) fleshy-fruited species are more prevalent in regions with younger floras than in regions with older ones.

## Materials and Methods

### Fruit Types of Angiosperms in China

We compiled a dataset on the fruit types of angiosperms in China from *Flora of China*^1^ and *Flora Reipublicae Popularis Bulgaricae*^2^. We classified all species into two categories based on their fruit types: species with fleshy fruits (including pome, berry, drupe, hesperidium, and pepo) and those with dry fruits (including legume, follicle, silique, and silicle, capsule, achene, nut, caryopsis, utricle, samara, and schizocarp). Our dataset contains fruit type data for 28,222 angiosperm species (including 5,604 fleshy-fruited species) from 2,998 genera and 250 families, accounting for 90.1% of angiosperms in China (Supplementary Table 1). Hybrid, aquatic, and cultivated species were excluded in.

### Distributions of Angiosperms in China

The distribution data of angiosperm species in China were compiled from published floras, atlases and peer-reviewed papers, including *Flora of China* (25 volumes), *Flora Reipublicae Popularis Bulgaricae* (126 volumes), *Higher Plants of China* (10 volumes, Fu, 1999–2005), the *Atlas of Woody Plants in China* (containing the distributions of all the 11,405 woody species; Fang et al., 2011; also see Wang et al., 2009), all provincial floras (149 volumes for 27 provinces), published floras and plant inventories at regional and local scales (42 volumes, such as floras of Karakorum-Kunlun Mountains, Nanling Mountains, Qinling Mountains and the Qinghai-Tibetan Plateau), and peer-reviewed papers on plant checklists and classification modifications from CNKI^3^. Species distributions were further examined and supplemented using recently published specimen records^4^. We standardized the Latin names of species from different data sources following the taxonomic treatment of *Flora of China*, which provides accepted names and synonyms of all seed plant species in China. The list of the data sources is included in Supplementary Appendix 2.

In order to improve the accuracy of species distribution data, we only compiled species distributions at county-level or finer scales (e.g., villages, towns, etc.) from published floras, atlases and papers, or those recorded as longitudinal and latitudinal coordinates of specimens. Most counties in China are relatively small, and the median area of all counties is 1,973.6 km^2^. It is noteworthy that the counties located in the Taklimakan Desert in southern Xinjiang and the alpine region in the northern Qinghai-Tibet Plateau are relatively large. To improve the accuracy of species distributions in these large counties, we divided each of them into 2–4 parts and species distributions in these counties were then downscaled to these relatively small geographical units using the information of the habitats and elevation ranges of species. Finally, more than 3.2 million county-level occurrence records of species were compiled.

To eliminate the potential bias of unequal area on subsequent analyses, we transformed the county-level distributions of species into gridded distributions with two different spatial resolutions: 50 × 50 km^2^ and 100 × 100 km^2^. Specifically, we intersected the county-level distributions of each species with the grids of different resolutions. When a grid cell was intersected with several counties where a species occurred, the total coverage of this grid cell by the county-level distribution of this species was the sum of all intersected parts of the counties with the presence of this species. The threshold for including a grid cell as presence is 1,106.66 km^2^ (which is the area of the smallest geographic unit, Hong Kong). Specifically, a grid cell with more than 1,106.66 km^2^ of its area covered by county-level distribution of a species was considered presence (see Supplementary Figure 3 for details). To reduce the potential influence of incomplete grid cells on the following analyses, we excluded the grid cells on country borders and along coasts that have less than half of their area being in China or on land (i.e., 1,250 km^2^ for 50 × 50 km^2^ grid cells; 5,000 km^2^ for 100 × 100 km^2^ grid cells). Finally, a total of 3,794 grid cells with the resolution of 50 × 50 km^2^ and 949 grid cells with the resolutions of 100 × 100 km^2^ were included in the following analyses. Similar methods have also been used to compile species distributions of woody plants in China (Wang et al., 2009, 2011).

### Geographic Patterns in the Proportions of Different Fruit Types

By integrating the fruit type data of all species with their distribution data, we calculated the proportions of fleshy-fruited species within each grid cell for all species combined, and for trees, shrubs, and herbaceous species separately. To evaluate the potential impact of spatial resolution on our results, we repeated all analyses using the data of the two spatial resolutions, and found that the results were highly consistent. Therefore, we presented the results based on 50 × 50 km grid cells in the main text, and those based on 100 × 100 km grid cells in Supplementary Table 3.

To eliminate the potential bias in the estimations of fruit type composition caused by small sample size of species, we included grid cells with more than five species in the following statistical analyses (see Supplementary Figure 2 for the species richness for each grid cells). With this threshold, no grid cells were excluded for the following analyses for all species and herbaceous species, nine for shrubs and 233 for trees. Most of these excluded grid cells are located in the Qinghai-Tibetan Plateau. To examine whether the choose of this threshold (i.e., five species) may influence our results, we repeated all analyses using grid cells with more than 10 species, and obtained highly consistent results. Therefore, we presented the results based on grid cells with more than five species in the main text and those based on grid cells with more than 10 species in Supplementary Tables 4,5.

### Growth Forms and Height of Angiosperms in China

A previous study showed that geographic patterns in the proportion of fleshy-fruited species differed between growth forms (Zhao et al., 2018). We therefore divided all species into three growth forms: herbaceous species, shrubs, and trees. Woody lianas were classified into shrubs, and herbaceous lianas and subshrubs were classified into herbaceous species. The growth form data of all species were obtained from *Flora of China* (accessed in April 2018) and *Flora Reipublicae Popularis Bulgaricae* (accessed in April 2018).

Data on mature plant height of woody species were obtained from Wang et al. (2019), and those of herbaceous species were compiled from *Flora of China* (accessed in April 2018) and *Flora Reipublicae Popularis Bulgaricae* (accessed in April 2018). Then, mean plant height per grid cell was calculated as the mean of the maximum mature height of all species within each cell and was used to evaluate the effects of plant height on the geographical variation in fruit type composition. Climbers, scandent shrubs and epiphytes were excluded in the analysis on plant height following Moles et al. (2009).

### Environmental Data

To assess the effects of climate on the geographic patterns of the proportion of fleshy-fruited species, we used the following six climatic variables (see Supplementary Table 2 for details): mean annual temperature (MAT), mean temperature of coldest quarter (MTCQ), mean precipitation of wettest quarter (MPWQ), Aridity Index (AI), temperature seasonality (TSN), and precipitation seasonality (PSN). AI reflects the level of aridity in a region, and TSN and PSN reflect the degree of climate seasonality within a year. These climate variables were grouped into three groups: temperature (MAT and MTCQ), precipitation (MPWQ and AI) and climate seasonality (TSN and PSN). MAT, MTCQ, MPWQ, TSN, and PSN with spatial resolutions of 1 × 1 km for the period 1970–2000 were obtained from the WorldClim dataset (Fick and Hijmans, 2017), and AI with a spatial resolution of 1 × 1 km was obtained from https://cgiarcsi.community/2019/01/24/global-aridity-index-and-potential evapotranspiration-climate-database-v2/.

Ecosystem primary productivity was represented by the following three variables: actual annual evapotranspiration (AET), net primary productivity (NPP), and gross primary productivity (GPP). AET is affected by both water availability and environmental energy, and has been used as a surrogate of ecosystem primary productivity in previous studies. AET was calculated using monthly average temperature and precipitation based on two different soil water storage capacity across different regions following Fang and Yoda (1990). NPP and GPP with a spatial resolution of 1 × 1 km were obtained from the Numerical Terra dynamic Simulation Group^5^. The mean for each variable within each grid cell of 50 × 50 km (or 100 × 100 km) was estimated with the zonal statistics tool in ArcGIS 10.0.

### Evolutionary History

To evaluate the effect of evolutionary history on the geographic patterns of the proportion of fleshy-fruited species, we used the dated genus-level phylogeny of seed plants in China constructed by Lu et al. (2018). This phylogeny was constructed using both plastid and mitochondrial genes, covering 2,665 Chinese vascular plant genera (ca. 92% of all genera known from China). In total, 138 fossil calibrations were used to calibrate the divergence time between different clades (see Lu et al., 2018 for more detail). Mean genus age was used to reflect effects of evolutionary age of floras on fruit type composition. We extracted the stem age of each genus and calculated the mean genus age for each grid cell as the mean of the ages of all genera within the grid cell.

### Statistical Analyses

We constructed univariate generalized linear models (GLMs) to explore the latitude patterns of the proportion of fleshy-fruited species and to evaluate the effects of each environment variable, mean plant height, and mean genus age on the proportion of fleshy-fruited species for all species, herbaceous species, shrubs, and trees separately. Following previous studies (Moles et al., 2009; Díaz et al., 2016), mean plant height was log-transformed and all explanatory variables were standardized before analyses. As the response variable was percentages of species within each grid cell, we used GLMs with binomial residuals and logit link for these regressions following previous studies (e.g., Chen et al., 2017; Zhao et al., 2018). McFadden’s *R*^2^ was calculated to assess the goodness-of-fit of each model (McFadden and Zarembra, 1973). The residuals of these models were all approximatively normally distributed, and contained no clear heteroscedasticity and non-linearity (Supplementary Figures 9,10), suggesting that binomial models performed well in fitting the data.

Spatial ecological data normally contain significant spatial autocorrelations, which could lead to overestimation of the degree of freedom of the residuals and hence the significance of regression models (i.e., Type I error; Lennon, 2000). In our analyses, we estimated the Moran’s I of the geographical patterns in the proportion of fleshy-fruited species using the “moran.test” function in the R package “spdep,” and found significant spatial autocorrelations (Supplementary Figure 6). To account for the effect of spatial autocorrelation on model significance, we tested the significance of all regression models using corrected degrees of freedom estimated by modified *t*-test (Dutilleul et al., 1993; see also Liu et al., 2017; Shrestha et al., 2018; Liu et al., 2019).

Further, we selected the best multivariate models explaining the patterns in proportions of fleshy-fruited species of each species group seperately using the Bayesian model averaging (BMA) method (Raftery et al., 1997). This method takes the uncertainty of the input models into consideration and provides a better predictive ability than traditional model selection methods (Wasserman, 2000), such as stepwise regression analysis. Previous studies (Hjort and Claeskens, 2003) and preliminary analyses indicate that model selection based on BMA may be biased by strong multicollinearity between predictors. Therefore, we selected the best single predictor of each environmental category with multiple variables (namely temperature, precipitation, climate seasonality, and ecosystem primary productivity) based on univariate GLMs for the following model selection. As both variables of climate seasonality (i.e., TSN and PSN) were not significant predictors of the proportion of fleshy-fruited species of any species group, this category was not included in the model selection. Finally, MAT representing environmental temperature, MPWQ representing precipitation, AET representing ecosystem primary productivity were selected to be included in multivariate model selection. Then we chose the top three models with the highest posterior probability (see Supplementary Figure 7 for all models selected). BMA model selection was conducted with the function “bic.glm” in the R package “BMA” (Raftery, 1995).

Then we used hierarchical partitioning method to compare the independent effects of different variables included in the best models using the function “hier.part” in R package “hier.part” (Chevan and Sutherland, 1991). In this analysis, the root-mean-square was used for the measure of the goodness of fit for binomial model. To further compare the effects of different variables on proportions of fleshy-fruited species, we conducted multiple spatial linear autoregressions with error model (SAR, Diniz-Filho et al., 2003; Hawkins et al., 2007; Supplementary Table 7), in which all variables included in the BMA best models were used as predictors of the proportion of fleshy-fruited species. For different species groups, the distances where the Moran’s I of the geographical patterns in the proportion of fleshy-fruited species became zero (Supplementary Figure 6) were used to define the neighborhood of each grid cell following previous studies. The Moran’s I of the residuals of SAR models were significantly reduced, suggesting that the SAR models have accounted for the spatial autocorrelation in the response variables. Spatial autoregressions were conducted using the R package “spatialreg” (Haining, 1990). The multicollinearity among variables was evaluated using the variance inflation factor (VIF), which was estimated by the function “vif” in R package “car” ver. 3.0-10 (Fox and Weisberg, 2018). The results indicated that the VIF for all the variables were less than 10 in all but one case (Supplementary Table 6).

All analyses were performed in R 3.4.3 (R Core Team, 2017).

## Results

### Patterns in Proportion of Fleshy-Fruited Species

Overall, fleshy-fruited species accounted for 19.9% of angiosperm species in China (see Supplementary Table 1). Trees had the highest proportion of fleshy-fruited species (50.5%), followed by shrubs (44.1%). Herbaceous species had the lowest proportion (5.94%) of fleshy-fruited species.

**FIGURE 1:**
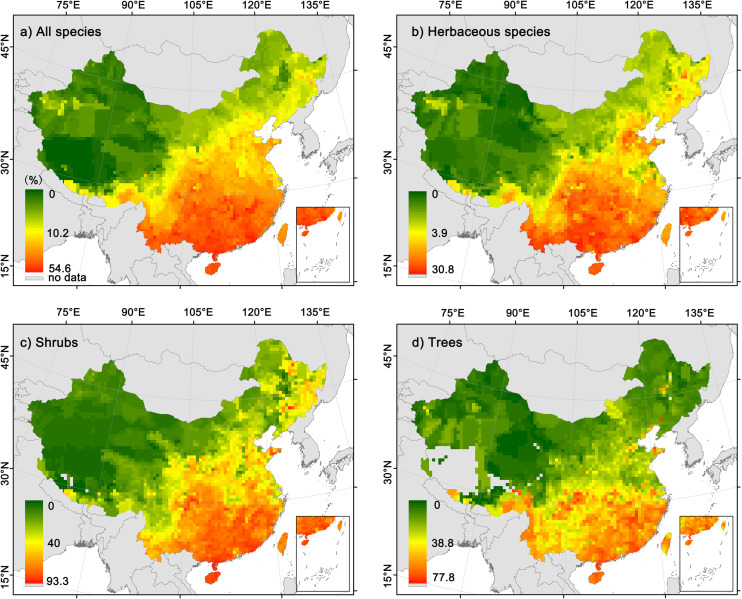
Geographic patterns in the proportions of fleshy-fruited species in local floras. **(a)** All angiosperm species; **(b)** herbaceous species; **(c)** shrubs; **(d)** trees.

For all species combined and different growth forms, the geographical patterns of the proportion of fleshy-fruited species were highly consistent (Figure 1). Specifically, the proportion of fleshy-fruited species significantly decreased with latitude (Supplementary Figure 4, all the *p*-values are less than 0.05), being the highest in southeast China but the lowest in northwest China. Although the geographical patterns in the proportion of fleshy-fruited species were consistent among different growth forms, the proportion of fleshy-fruited species for herbaceous species was generally much lower than those of trees and shrubs in most regions.

### Effects of Different Factors on Proportion of Fleshy-Fruited Species

**FIGURE 2:**
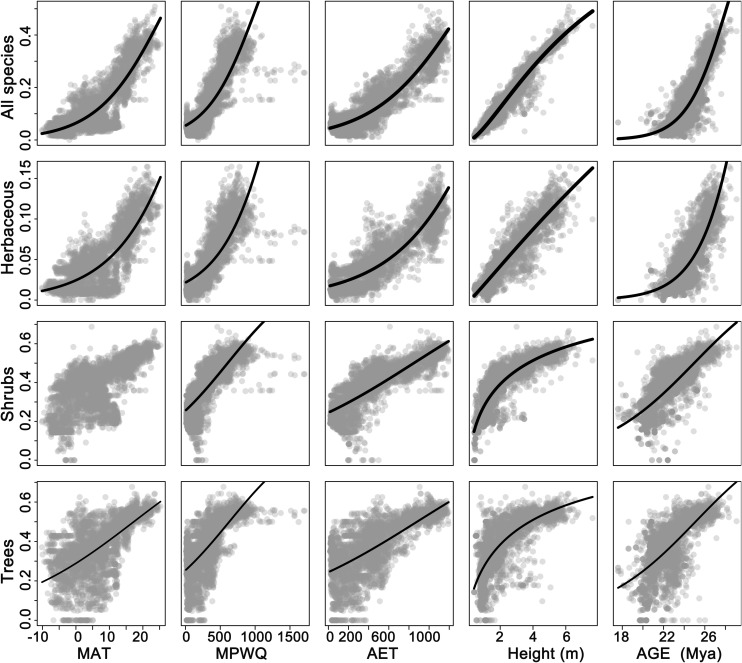
The relationships between proportions of fleshy-fruited species per grid cell and different variables. The five columns from the left to the right represent mean annual temperature (MAT), mean precipitation of wettest quarter (MPWQ), actual annual evapotranspiration (AET), mean plant height, and mean genus age, respectively. The four rows from top down represent all species, herbaceous species, shrubs, and trees, respectively.

Univariate models indicated that among all factors tested, mean plant height per grid cell and AET representing ecosystem primary productivity (Rosenzweig, 1968; Qian, 2013) were consistently the strongest and second strongest predictors of the geographic patterns in the proportion of fleshy-fruited species for all species combined and different growth forms (Table 1). Specifically, the proportion of fleshy-fruited species was all significantly positively associated with mean plant height per grid cell and AET. Among variables representing contemporary climate, MAT and MTCQ had comparable effects on the proportion of fleshy-fruited species (Table 1), while MPWQ had stronger effects than AI. The effects of climate seasonality were not significant for any species group (Table 1). Mean genus age per grid cell was positively correlated with the proportion of fleshy-fruited species for all species combined and for the three different growth forms separately (*p* < 0.05), while its explanatory power was weaker than those of mean plant height per grid cell and AET (Figure 2 and Table 1).

**TABLE 1:** The slopes and explanatory power (*R*^2^) of the univariate models between proportions of fleshy-fruited species and variables representing contemporary environment, mean plant height and mean genus age.

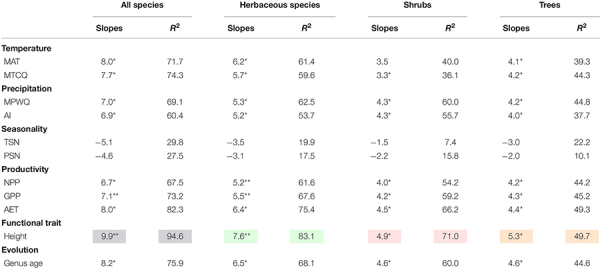

Generalized linear models (GLMs) with binomial residuals and logit link was used to model these relationships. The slopes are multiplied by 10 for easier comparison. The strongest explanatory variable for each growth form is highlighted by different color. Significance levels of each explanatory variable: *p < 0.05, **p < 0.01.

Bayesian model selection (BMA) indicated that the mean plant height per grid cell was selected as the single predictor or one of the predictors in all of the best models for all species combined and different growth forms except the top best model for trees (Table 2). In contrast, AET was selected in the top best models for shrubs and trees, and in the second best models for herbaceous species and trees. Among climate variables, MAT was selected in the top best model for trees and MPWQ was selected in the third best model for shrubs. The mean genus age per grid cell was selected in the second or the third best models for all species combined, shrubs and trees.

**TABLE 2:** The best models explaining the proportions of fleshy-fruited species for all species and different growth forms selected by Bayesian model averaging (BMA) method.

Growth forms	Models		Posterior probability	*R*^2^ (%)	AIC	*p*-Value
All species	Model 1	Height	0.537	94.2	1,250.1	<0.01
	Model 2	Height + age	0.255	96.2	1,255.6	<0.01
	Model 3	Height + AET	0.151	95.8	1,254.7	<0.01
	Combined	Height + AET + age	–	96.5	1,257.9	<0.01
Herbaceous	Model 1	Height	0.949	83.1	382.4	<0.01
	Model 2	Height + AET	0.051	85.5	384.6	<0.01
	Combined	Height + AET	–	85.5	384.6	<0.01
Shrubs	Model 1	AET + height	0.675	75.6	3,506.3	0.019
	Model 2	Age + height	0.227	74.9	3,531.7	0.017
	Model 3	MPWQ + height	0.098	74.2	3,544	0.018
	Combined	Height + AET + age + MPWQ	–	76.2	3,506.3	0.016
Trees	Model 1	AET + MAT	0.228	53.3	3,342.1	0.024
	Model 2	AET + height	0.226	53.3	3,374.1	0.021
	Model 3	Height + age	0.186	53.1	3,384	0.026
	Combined	AET + MAT + age + height	–	55.1	3,329	0.028

*The posterior probability, explanatory power (*R*^2^), AIC, and *p*-value of each selected model are shown.*

**FIGURE 3:**
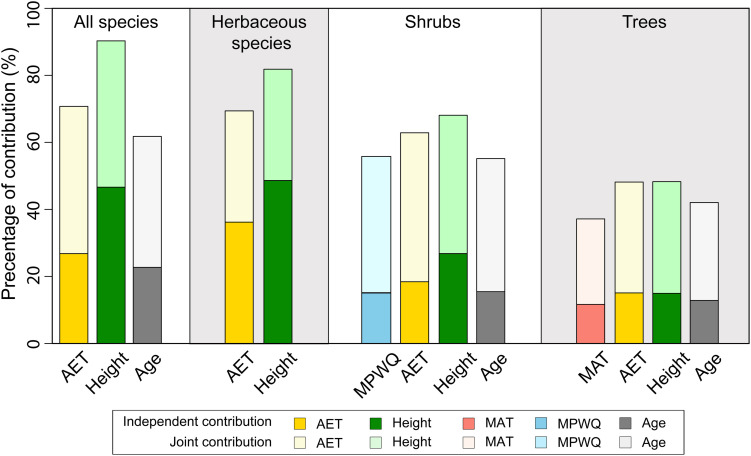
The independent (darker colors) and joint (lighter colors) contributions of different variables included in the best models explaining the proportions of fleshy-fruited species per grid cell selected by Bayesian model averaging (BMA) method. Hierarchical partitioning method was conducted for all species, herbaceous species, shrubs, and trees separately.

The hierarchical partitioning analyses indicated that mean plant height per grid cell had higher total and independent effects on proportion of fleshy-fruited species than AET for all species combined, herbaceous species and shrubs, but not for trees (Figure 3). In all cases, the total and independent effects of mean genus age were generally lower than those of both mean plant height and AET (Figure 3). For shrubs and trees, climate variables including MPWQ and MAT had relatively lower total and independent effects than mean plant height and AET (Figure 3).

## Discussion

In this study, we demonstrated the geographic patterns in the proportion of fleshy-fruited angiosperm species in China and explored the drivers shaping these patterns. We found that mean plant height per grid cell representing vegetation height and AET representing ecosystem primary productivity were the dominant drivers of the geographical patterns in the proportion of fleshy-fruited species, but their relative effects significantly varied across growth forms. Moreover, the relative importance of mean plant height per grid cell decreased from herbaceous species to shrubs and trees.

### Strong Correlation Between Vegetation Height and Fleshiness

Mean plant height per grid cell, which is in general associated with vegetation height of a region, had the strongest positive effect on the geographic pattern in the proportion of fleshy-fruited species for all species combined, herbaceous species and shrubs. These results support our hypothesis that fleshy-fruited species are more prevalent in regions dominated by taller plant species and also corroborate that the evolution of fleshy fruit may be strongly associated with the evolution of vegetation structure at large scales (Tiffney, 1984; Bolmgren and Eriksson, 2005, 2010; Eriksson, 2008; Bonser, 2013).

The positive association between the proportion of fleshy-fruited species and mean plant height per grid cell are consistent with previous studies based on both fossil records and contemporary community data. For example, from a paleontological perspective, Kovar-Eder et al. (2013) studied the fossil records in the Eocene and Neogene from Europe and showed that dense vegetation with taller species (such as paratropical forests and broad-leaved evergreen forests) had higher proportion of fleshy-fruited species compared with less dense communities dominated by shorter species (e.g., sub-humid forests). Moreover, based on data of different contemporary communities in northern China, Yu et al. (2017) found that vegetation with taller plant species tends to contain more fleshy-fruited species. Similarly, Knorr et al. (2012) compiled forest community data from 13 mountains in both China and Japan and found that fleshy-fruited species are more prevalent in tropical and subtropical broad-leaved evergreen forests than in broad-leaved deciduous forests and broad-leaved monsoon forests. Tropical and subtropical broad-leaved evergreen forests normally have relatively higher and more closed canopies than broad-leaved deciduous forests and broad-leaved monsoon forests, which might be one of the reasons for the difference in the prevalence of fleshy-fruited species between these forest types. In addition, the vegetation height may also be associated with variations in proportion of woody plants. In general, forests in tropical and subtropical regions tend to have more woody plants which have more fleshy-fruited species than herbaceous species. Together, these findings support the strong association between the variation in the height of vegetation and the proportions of fleshy-fruited species in floras across both space and time for angiosperms.

### Effects of Contemporary Environment on Fruit Type Composition

Our results suggest that AET was selected in most of the best models for all species and the three different growth forms, and had significant positive effects on the proportion of fleshy-fruited species (Table 1 and Figure 3). As AET has been widely used as a surrogate of ecosystem primary productivity in previous studies (Tang et al., 2009), this result supports our hypothesis and suggests that ecosystem primary productivity may be one of the major determinants of the prevalence of fleshy-fruited species in communities. This is consistent with previous findings that high primary productivity could benefit the development of fleshy fruits due to the high energy consumption (e.g., respiratory climacteric) during the complex maturation process of fleshy fruits (Coombe, 1976; Seymour et al., 2013). An analysis based on 1,247 woody communities indicates that stand biomass of a community is positively associated with its primary productivity (Michaletz et al., 2014). High biomass and high primary productivity could supply enough energy support for the development of fleshy fruits, which may explain the significant strong independent effect of primary production (as represented by AET) on the proportion of fleshy-fruited species.

Moreover, primary productivity could also indirectly influence the composition of fruit types via its effects on plant height. Recent studies indicate that primary productivity is significantly associated with plant height in eastern Asia (Wang et al., 2019) and at the global scale (Zhang et al., 2016). Tall plants and canopies tend to generate more shaded environment favoring fleshy-fruited species as shown in previous studies (Leishman and Westoby, 1994; Bolmgren and Eriksson, 2005; Knorr et al., 2012; Lagomarsino et al., 2016).

Our results suggest that variables representing environmental temperature and precipitation were not selected in the best models explaining the proportion of fleshy-fruited species for all species combined and for herbaceous species (Figure 3 and Tables 1, 2). In addition, although climate variables including MPWQ and MAT may also influence the geographical variations in the proportion of fleshy-fruited species of shrubs and trees, respectively, their effects were weaker than the effect of AET and mean plant height. These findings suggest that temperature and precipitation variables may not be the dominant drivers of geographical patterns in the prevalence of fleshy-fruited species. This might be due to the fact that neither temperature nor precipitation alone could reflect the actual amount of energy and water that plants use for growth (Moles et al., 2014). These findings are in contrast with previous studies on the determinants of fruit type compositions (Chen et al., 2017; Zhao et al., 2018). For example, a recent study on the geographical pattern in the prevalence of fleshy-fruited angiosperm species in Australia (Chen et al., 2017) indicated that precipitation dominated the geographical variations in the proportion of fleshy-fruited species. Similarly, another study in China (Zhao et al., 2018) concluded that mean annual precipitation dominated the geographical variation in the proportion of fleshy-fruited species for woody species, but environmental temperature dominated that of herbaceous species. It is noteworthy that these studies (Chen et al., 2017; Zhao et al., 2018) did not compare the effects of environmental temperature and precipitation with those of mean plant height and ecosystem primary productivity, and hence failed to identify the dominant roles of mean plant height and ecosystem primary productivity in shaping the geographical variations in the prevalence of fleshy-fruited species. Together, our results cast doubt on recent conclusions that environmental temperature and precipitation dominate the prevalence of fleshy-fruited species, and suggest that the role of mean plant height and ecosystem primary productivity (AET) cannot be ignored in understanding the determinants shaping patterns of fruit type composition.

### Effects of Different Factors Vary Among Growth Forms

Both the BMA selection and the hierarchical partitioning analysis suggest that the relative importance of mean plant height per grid cell in shaping the geographical patterns of the proportion of fleshy-fruited species decreased from herbaceous species and shrubs to trees (Figure 3). Specifically, the independent effect of mean plant height was higher than those of AET for herbaceous species and shrubs, but became comparable to those of AET for trees. Moreover, mean plant height per grid cell was not even selected in the top best model explaining proportion of fleshy-fruited species for trees. These results suggest that the effect of mean plant height on the maintenance of fleshy-fruited species is weaker for taller species than for shorter ones (also see Supplementary Figure 5). In forests where mean plant height is tall, herbaceous species and shrubs generally live in closed and shaded understory environment where animal dispersal is the favorable way for seed dispersal. It has been suggested that fleshy fruit is an adaptation of plants to attract animal dispersers (Jordano, 1995; Eriksson, 2008, 2016). For example, a recent study based on 3,200 plant species in Cerrado biome showed that most understory zoochory species produced fleshy fruits (Kuhlmann and Ribeiro, 2016). This may partly explain why plant height has strong effect on the proportions of fleshy-fruited species for herbaceous and shrub species.

In contrast to herbaceous and shrub species, trees generally dominate the canopy, and may be less dependent on animal dispersal but more on wind dispersal. A recent study indicated that taller tree species tend to be wind-dispersed rather than animal-dispersed (Thomson et al., 2018). For instance, the tall species in *Populus* take better advantage of wind power, and have a better adaptation to wind dispersal (Zhao et al., 2016). Therefore, different growth forms may have different strategies to improve seed dispersal and producing fleshy fruits is not the only way for far seed dispersal and high offspring survival for tree species.

### The Effect of Evolutionary Age on Fruit Type Composition

We found that older floras have more fleshy-fruited species than younger floras, which is in contrast with our hypothesis (Table 1, also see Supplementary Figure 5). Previous studies found that fleshy-fruited species, often associated with animal dispersal and seed defense (Mack, 2000; Bolmgren and Eriksson, 2005), tend to have higher competitive ability and fitness and hence lower extinction rates than dry-fruited species. For example, a study found that lineages with fleshy fruits had higher evolutionary success than those with dry fruits (Vamosi and Vamosi, 2004). Similarly, Larson-Johnson (2016) found that endozoochory lineages including those with fleshy fruits (e.g., genus *Morella* of Myricaceae) tend to have lower extinction rate than wind-dispersed lineages. Moreover, previous studies have also demonstrated that fleshy fruits are strongly associated with woody growth form (Willson et al., 1989) and large seed mass (Leishman and Westoby, 1994; Butler et al., 2007), which normally have low evolutionary rates (Smith and Donoghue, 2008; Lanfear et al., 2013; Igea et al., 2017). The low extinction rate of fleshy-fruited species may lead to better survival of old fleshy-fruited species than dry-fruited ones during the evolutionary history, and hence a positive correlation between mean lineage age and the proportion of fleshy-fruited species in floras.

Alternatively, the positive correlation between the proportion of fleshy-fruited species and the evolutionary age of floras could also be because they both respond to similar environmental gradients. Subtropical southeastern China has high primary productivity, and has remained similar climate since early Eocene (Lu et al., 2018). The high primary productivity in this region may have led to high proportions of fleshy-fruited species in floras, while the stable climate may have led to the preservation of old lineages. These processes may also lead to the positive correlation between proportions of fleshy-fruited species and mean genus age of local floras. Further evaluations are needed to better understand the mechanisms underlying the positive association between proportions of fleshy-fruited species and mean lineage age within floras.

## Conclusion

Here we compared the relative effects of contemporary climate, ecosystem primary productivity, mean plant height, and genus age on the geographic patterns in proportions of fleshy-fruited species using a large dataset of fruit types and distributions of angiosperm species in China. Our results suggest that mean plant height and actual evapotranspiration representing ecosystem primary productivity were the strongest determinants of the geographical variations in the proportion of fleshy-fruited species. However, the relative importance of these factors varies significantly across plant growth forms. From herbaceous species to shrubs and trees, the relative importance of mean plant height decreases. In addition to these two factors, environmental temperature and precipitation may also contribute to the variation in the proportion of fleshy-fruited species of trees and shrubs, respectively. Compared with ecosystem primary productivity and mean plant height, mean genus age of floras had consistently weaker effects. These results improve our understanding of the mechanisms underlying the geographical patterns in fruit type composition, and suggest that biotic and environmental factors and evolutionary age of floras jointly shape the pattern in reproductive traits of plants. Given the variations in climate and floras across different continents, more studies are needed to test the generality of our findings in different regions and at larger spatial scales.

## Data Availability Statement

The original contributions presented in the study are included in the article/Supplementary Material, further inquiries can be directed to the corresponding author/s.

## Author Contributions

ZW, HZ, and TL designed the research. TL, YL, and YW collected the trait data. AL and TL collected the species distribution data. TL, HC, and YW performed the analyses. All authors discussed the results. TL, ZW, YW, and SP led the writing with contributions from all co-authors.

## Conflict of Interest

The authors declare that the research was conducted in the absence of any commercial or financial relationships that could be construed as a potential conflict of interest.

## Footnotes

1 http://www.efloras.org/flora_page.aspx?flora_id=2 (accessed in April 2018).

2 http://www.iplant.cn/frps (accessed in April 2018).

3 https://www.cnki.net/ (accessed in April 2018).

4 see http://www.papc.cn/ and http://www.nsii.org.cn/ (accessed in April 2018).

5 http://www.ntsg.umt.edu/project/modis/mod17.php (accessed in April 2018).
