# Supplementary material for: Effects of Climate, Plant Height, and Evolutionary Age on Geographical Patterns of Fruit Type
Source: Front Plant Sci. 2021 Mar 16;12:604272. doi: 10.3389/fpls.2021.604272 (PMC8007967; doi:10.3389/fpls.2021.604272)
Supplement: Supplementary file 1 [file Data_Sheet_1.docx]

Supplementary Material

# Supplementary Figures and Tables

## Supplementary Figures


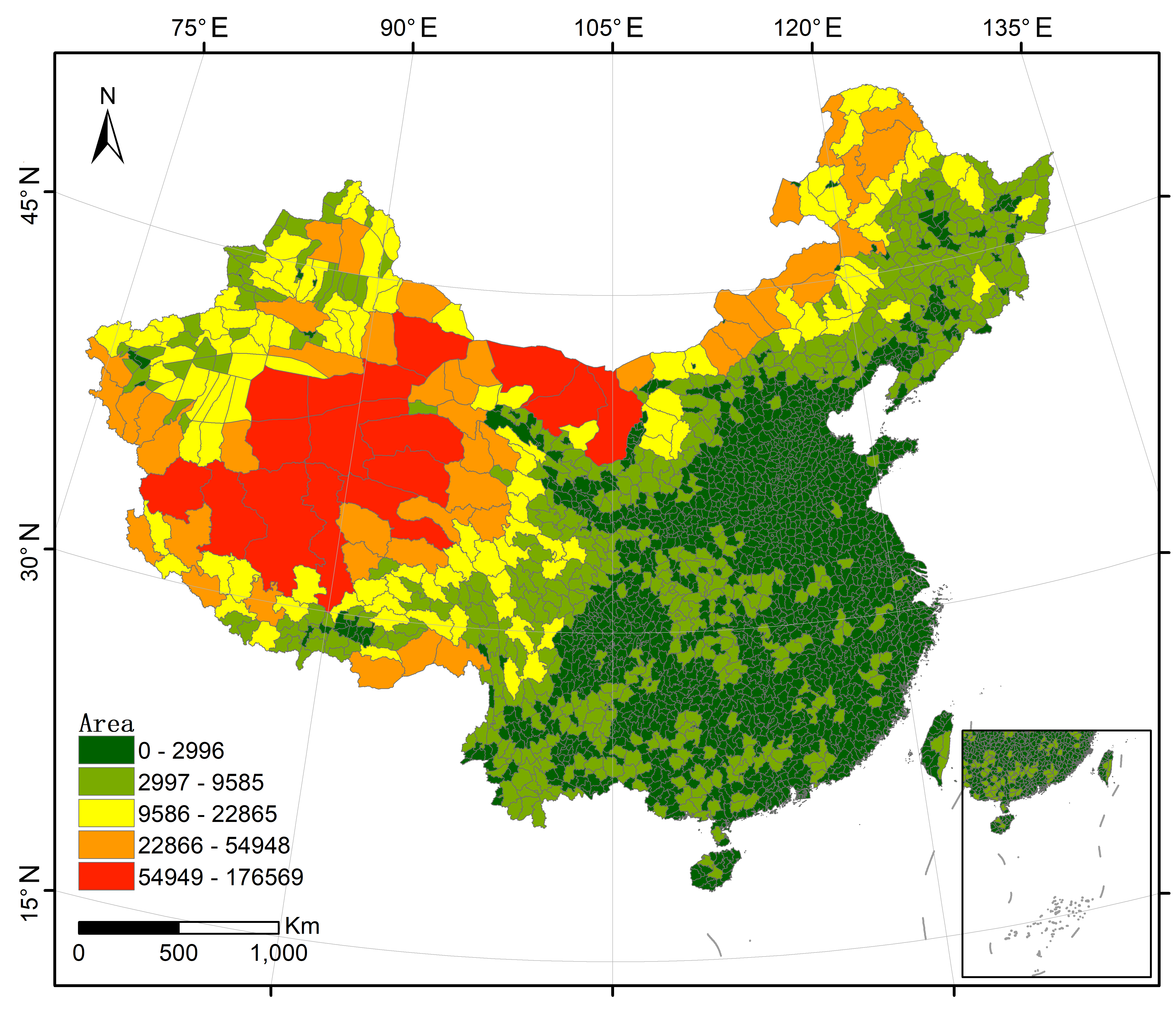


**Figure S1** The area of grid cells used for species distribution data.

**
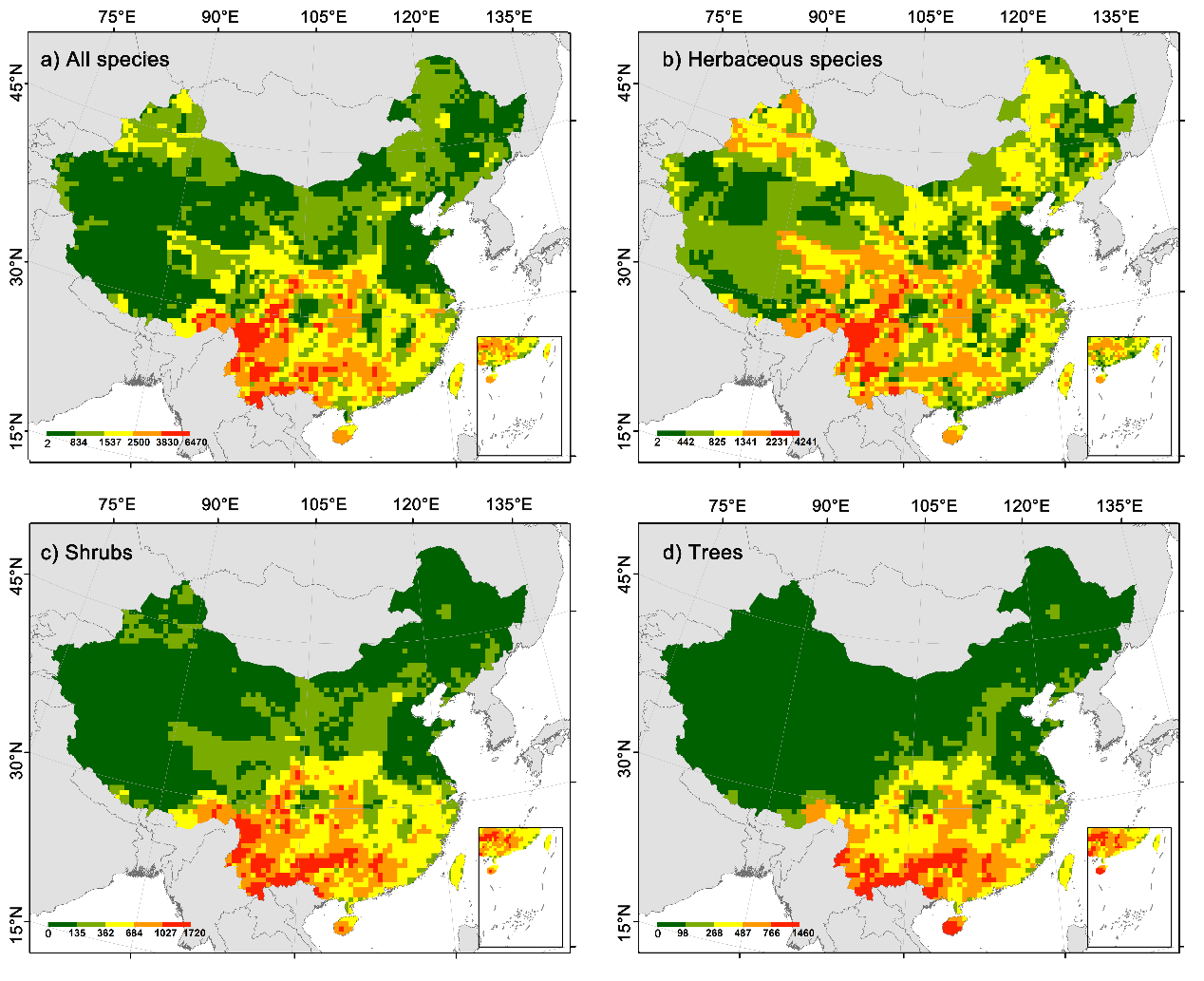
**

**Figure S2** The patterns in species richness for the species used in our analyses. a) all species; b) herbs; c) shrubs; d) trees.


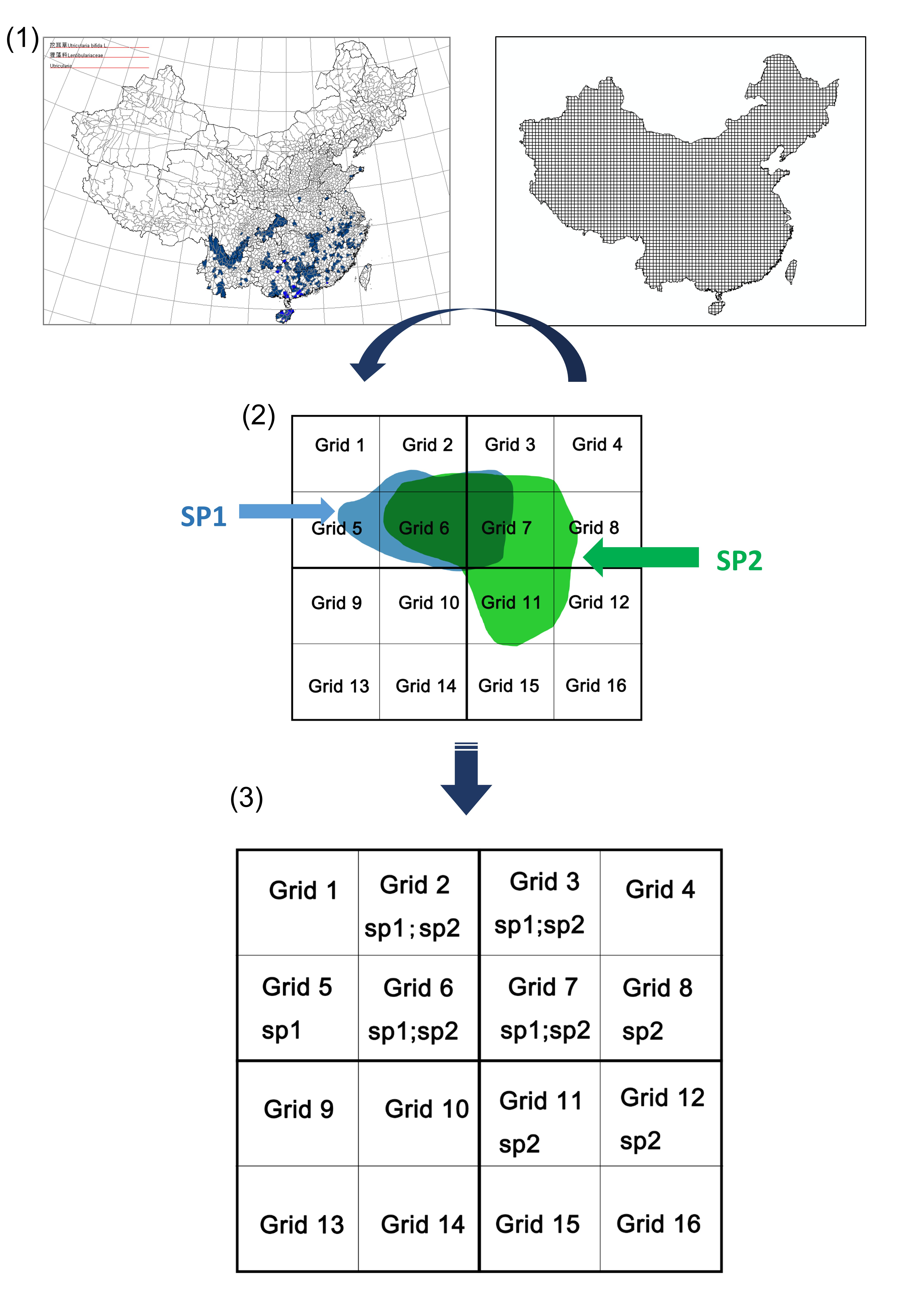


**Figure S3** Detailed flow diagram for steps on the transition from county-scale records to 50 * 50 km grid cells.

**
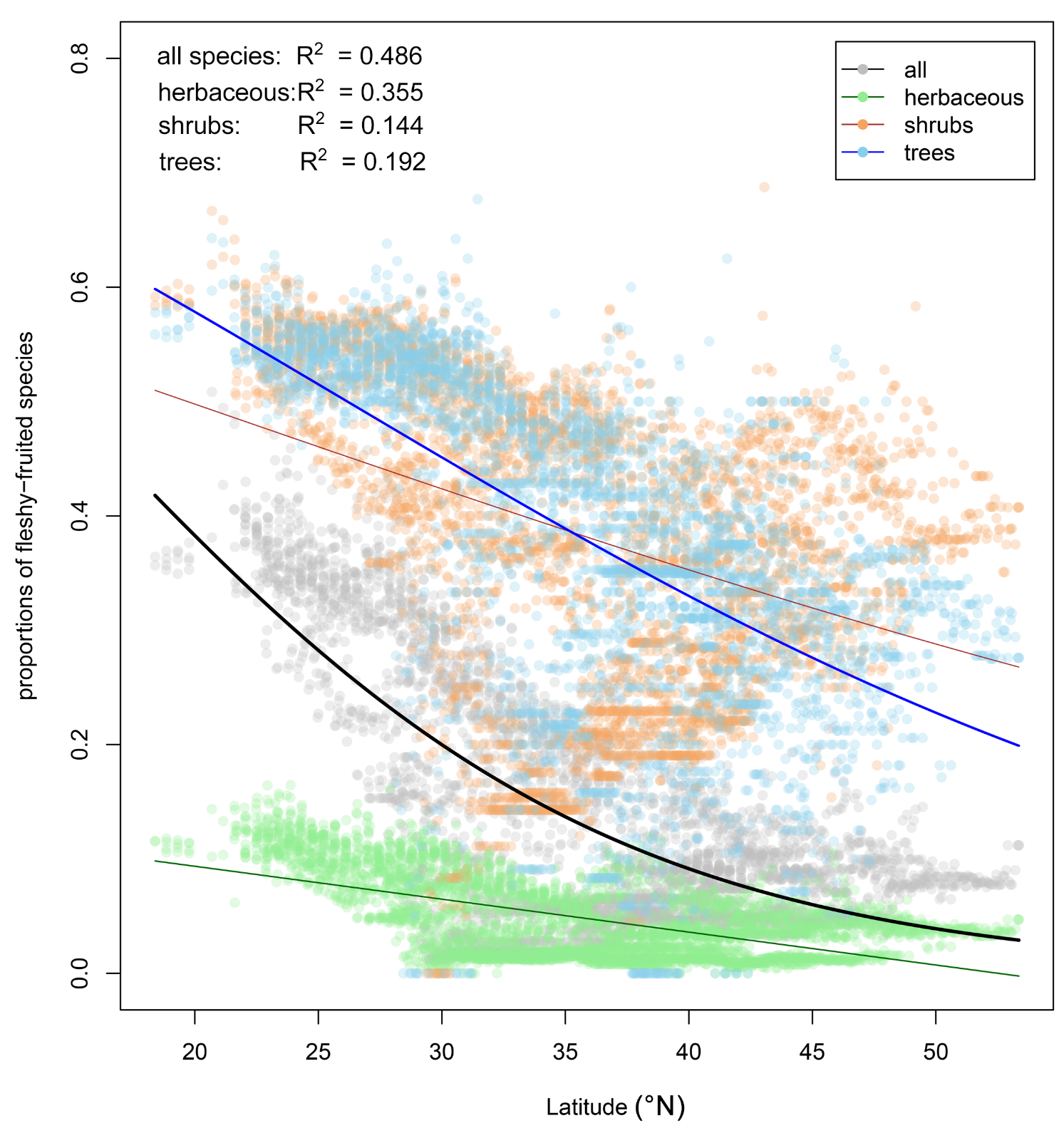
Figure S4** The relationship between proportion of fleshy-fruited species and latitude.

**
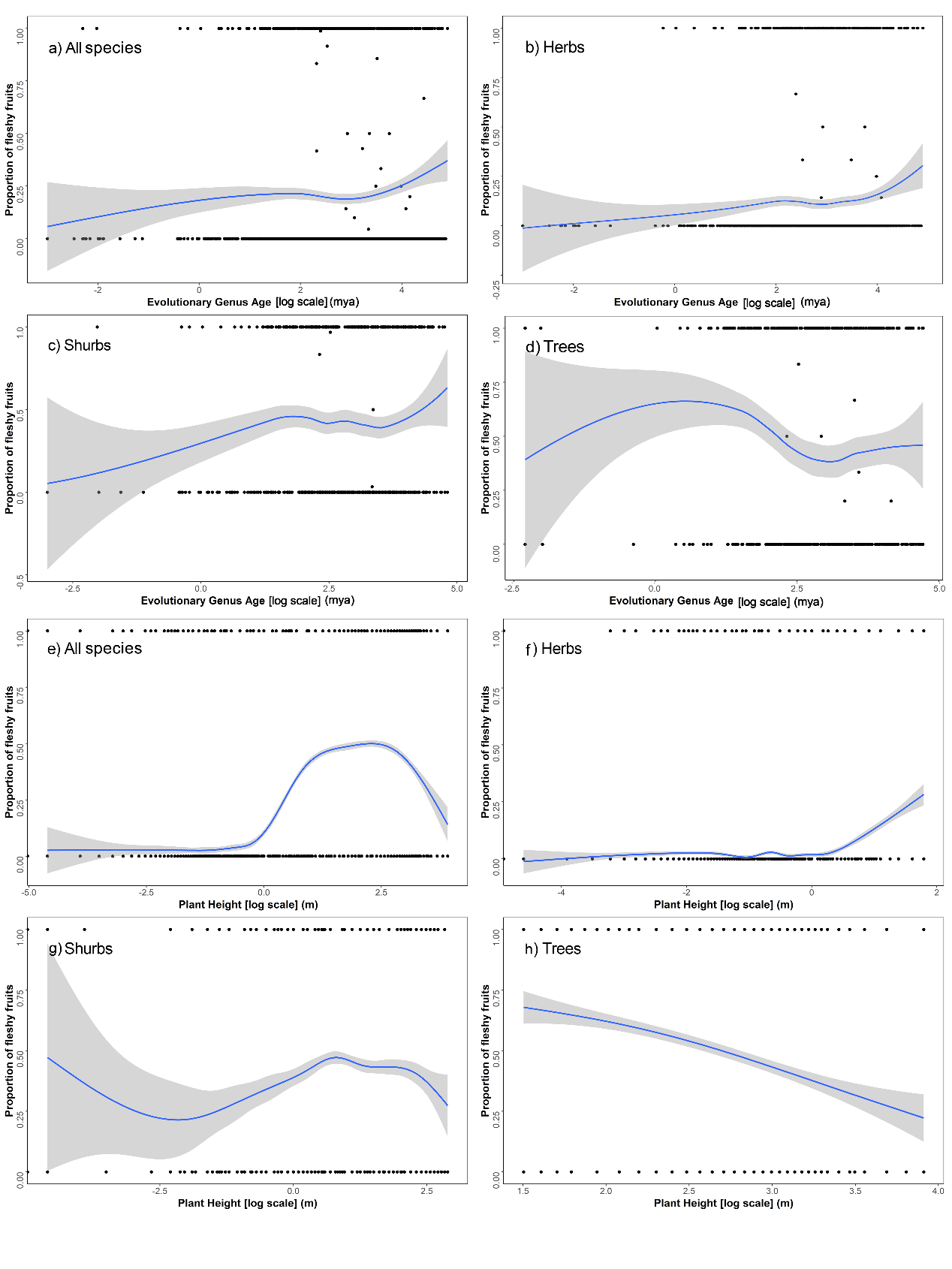
**

**Figure S5** The relationships between fruit type (fleshy-fruited: 1; dry-fruited: 0) and plant height at species level and between proportions of fleshy-fruited species within genera and genus age. The blue lines are estimated with GAM and are used to demonstrate the non-linear relationships between different variables. The grey areas show the 95% confidence interval of the GAM regression lines.


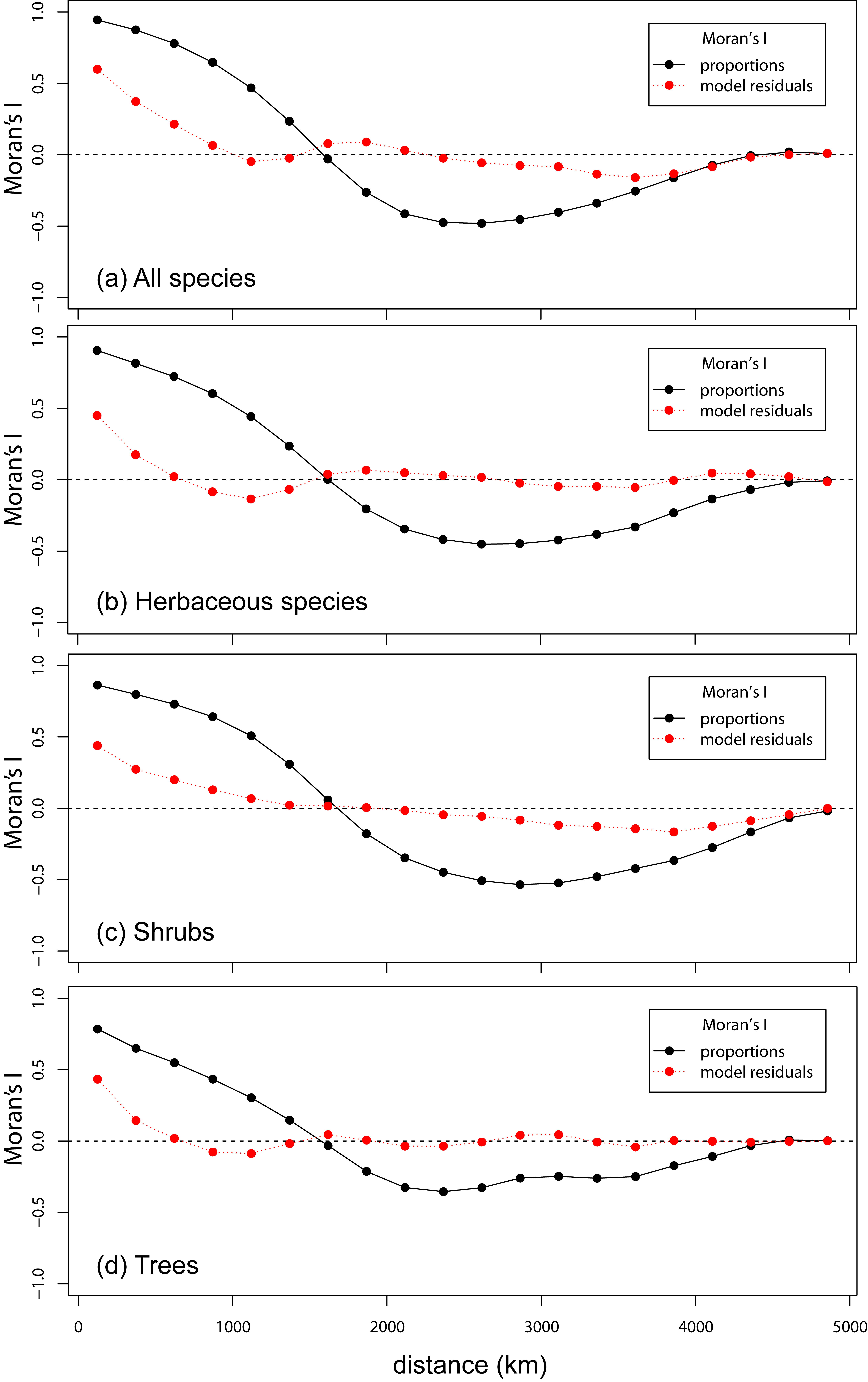


**Fig. S6** Spatial autocorrelogram of the geographical patterns in the proportion of fleshy-fruited species and the residuals of the best multiple autoregression models explaining the proportion of fleshy-fruited species evaluated by Moran’s I. (a) all species; (b) herbaceous specie; (c) shrubs; (d) trees.





**Figure S7** All the models selected by Bayesian model averaging (BMA) method. (a) all species; (b) herbaceous specie; (c) shrubs; (d) trees.


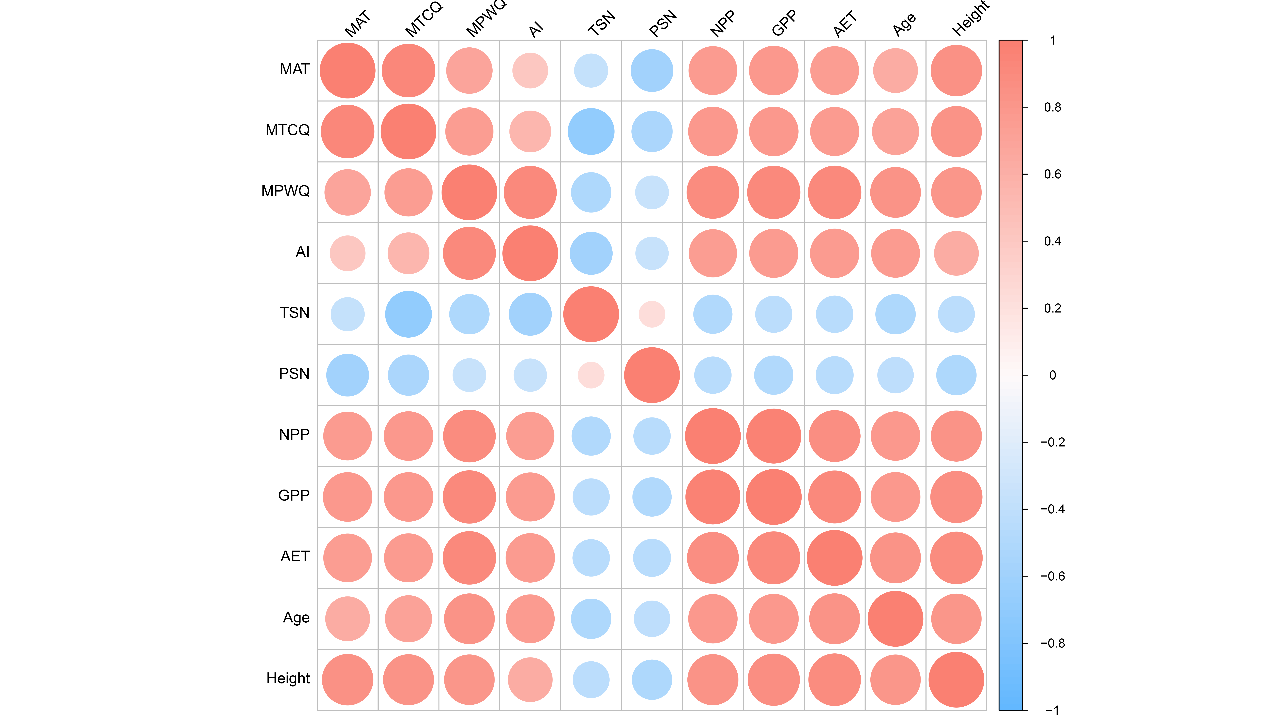


**Figure S8** Correlation coefficient among all the variables for each grid cells.

**Fig. S9** The frequency distributions of the residuals of each regression model (the first five columns) and the observed proportion of fleshy-fruited species (the last column).


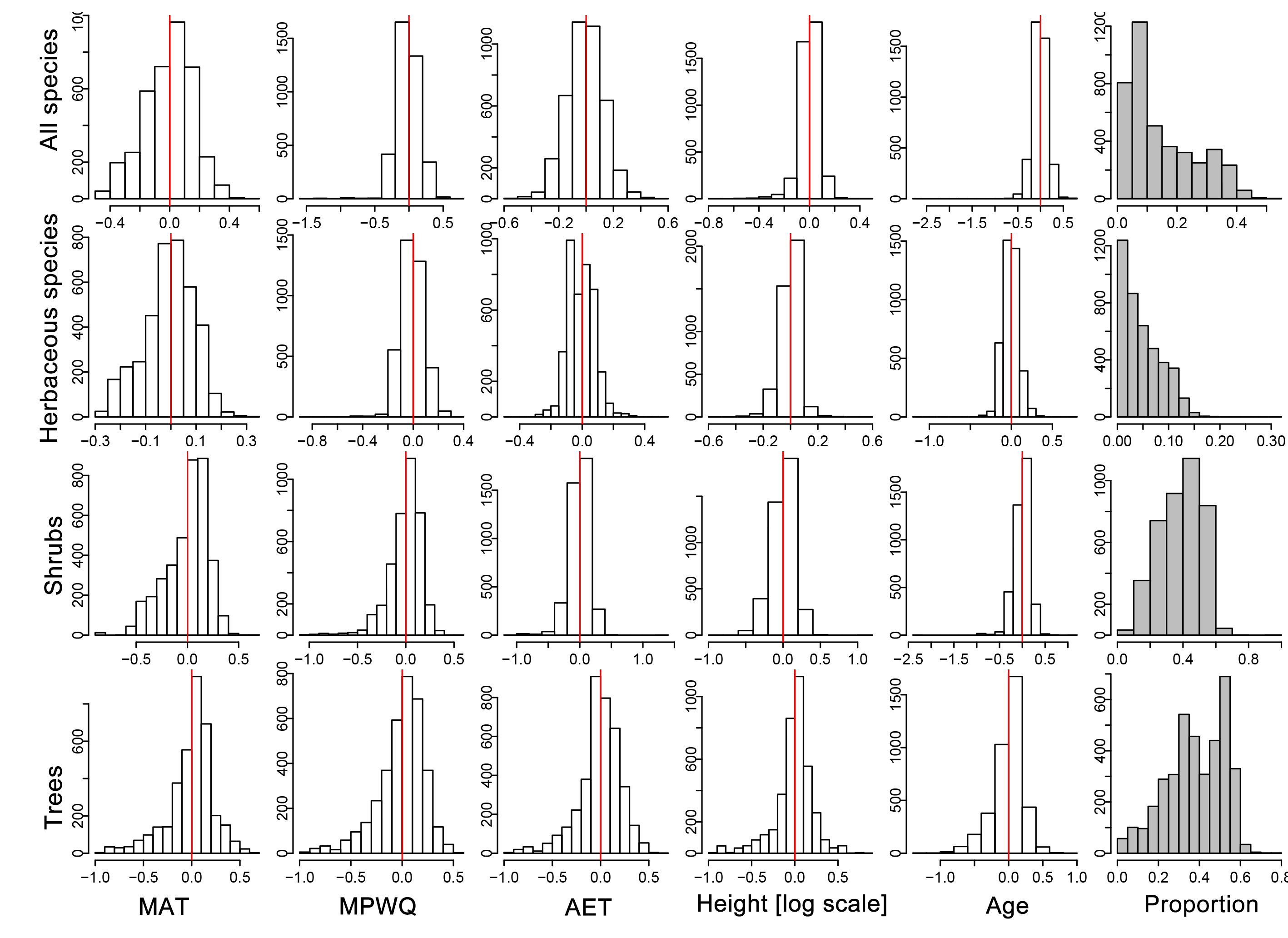

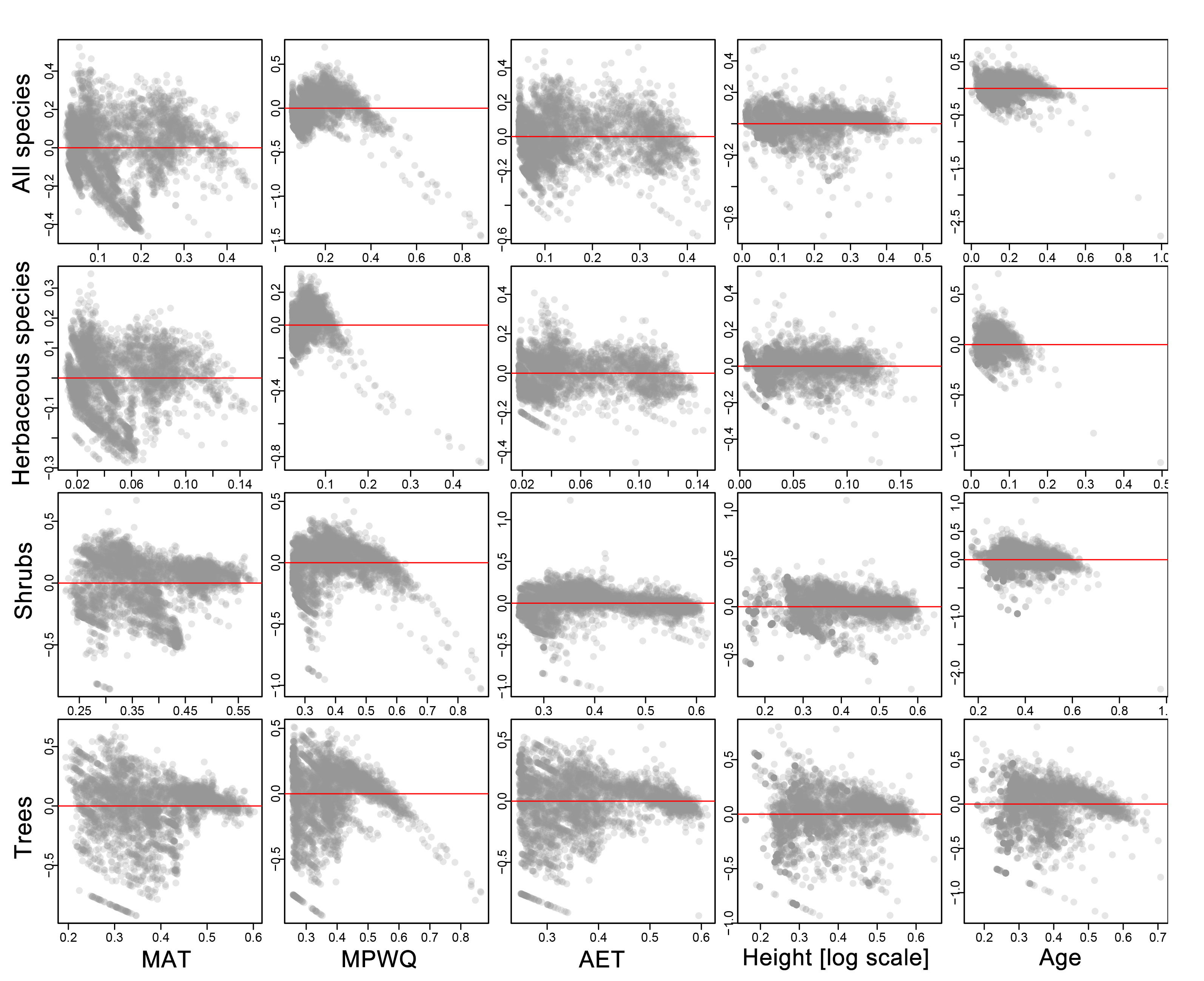


**Fig. S10** The relationship between the deviance residuals and the fitted values for each regression model between the proportion of fleshy-fruited species and predictors. MAT, mean annual temperature; MPWQ, mean precipitation of the wettest quarter; AET, actual annual evapotranspiration; Height, mean plant height per grid cell; Age, mean genus age per grid cell.

## Supplementary Tables

**Table S1** The fruit type spectrum of Chinese flowering plants.

| Life forms | Dry-fruited species | | Fleshy-fruited species | | All species |
| --- | --- | --- | --- | --- | --- |
|  | No. | % | No. | % | No. |
| Herbaceous species | 17426 | 94.06 | 1101 | 5.94 | 18526 |
| Shrubs | 3410 | 55.89 | 2691 | 44.11 | 6101 |
| Trees | 1781 | 49.55 | 1813 | 50.45 | 3594 |
| Total number | 22618 | 80.14 | 5604 | 19.85 | 28222 |

**Table S2** Variables and ecological meaning.

| acronyms | Full Variable name | Ecological meaning | Data sources |
| --- | --- | --- | --- |
| **Temperature** |  |  |  |
| MAT | Mean annual temperature (℃) | General environmental energy | World clim-Bio1 |
| MTCQ | Mean temperature in coolest quarter (℃) | Cold stress | World clim-Bio11 |
| **Precipitation** |  |  |  |
| MPWQ | Mean precipitation in wettest quarter (mm) | Water available in growing season | World clim-Bio16 |
| AI | Arid Index | Water shortage | Trabucco, Antonio; Zomer, Robert (2019) |
| **Seasonality** |  |  |  |
| TSN | Temperature seasonality | Seasonal disparity in temperature | World clim-Bio4 |
| PSN | Precipitation seasonality | Season al disparity in precipitation | World clim-Bio15 |
| **Productivity** |  |  |  |
| NPP | Net primary productivity | carbon store during photosynthesis, which effected by climate and plant traits | MODIS GPP/NPP Project (MOD17) |
| GPP | gross primary productivity | summation of carbon store and respiration consumption | MODIS GPP/NPP Project (MOD17) |
| AET | Actual evapotranspiration (mm) | water-energy balance | Fang & Yoda (1990) |

**Table S3** The slopes and explanatory power (R^2^) of the univariate models between proportions of fleshy-fruited species and variables representing contemporary environment, functional traits and genus age for 100 * 100 km grid cells. The slopes are multiplied by 10 for easier comparison. The strongest explanatory variable for each growth form is highlighted by different color. Significance levels of each explanatory variable: *, p < 0.05, **, p < 0.01.

|  | All species | | Herbaceous species | | Shrubs | | Trees | |
| --- | --- | --- | --- | --- | --- | --- | --- | --- |
|  | Slopes | R^2^ | Slopes | R^2^ | Slopes | R^2^ | Slopes | R^2^ |
| **Temperature** |  |  |  |  |  |  |  | |
| MAT | 7.1* | 73.9 | 5.9* | 63.9 | 3.2 | 40.1 | 3.6* | 43.5 |
| MTCQ | 6.8* | 76.4 | 5.5* | 62.6 | 3.0 | 37.5 | 3.6* | 48.1 |
| **Precipitation** |  |  |  |  |  |  |  |  |
| MPWQ | 6.3* | 69.3 | 5.3* | 67.1 | 4.1* | 65.6 | 3.5* | 43.0 |
| AI | 3.9 | 32.3 | 3.1 | 26.8 | 2.6 | 27.5 | 2.6* | 24.9 |
| **Seasonality** |  |  |  |  |  |  |  |  |
| TSN | -4.6 | 31.0 | -3.4 | 21.7 | -1.5 | 9.0 | -2.4 | 21.6 |
| PSN | -4.1 | 27.8 | -3.1 | 19.2 | -2.0 | 15.7 | -1.9 | 12.8 |
| **Productivity** |  |  |  |  |  |  |  |  |
| NPP | 6.3* | 69.7 | 5.3* | 65.8 | 3.9* | 57.0 | 3.7* | 45.8 |
| GPP | 6.6** | 75.3 | 5.6** | 71.8 | 4.0* | 62.2 | 3.8* | 46.3 |
| AET | 7.2** | 84.5 | 6.4* | 82.4 | 4.3* | 74.6 | 3.6* | 46.4 |
| **Functional trait** | |  |  | 7.4 |  | 4.4 |  | 4.4 |
| Height | 8.6** | 97.1  78.5 | 7.4** | 89.5 | 4.4* | 73.9 | 4.4* | 56.7 |
| **Evolution** |  |  |  |  |  |  |  |  |
| Genus Age | 7.2* | 78.5 | 6.2* | 73.8 | 4.2* | 67.1 | 3.6* | 45.0 |

**Table S4** The slopes and explanatory power (R^2^) of the univariate models between proportions of fleshy-fruited species and variables representing contemporary environment, functional traits and genus age for grid cells with more than 10 species. The slopes are multiplied by 10 for easier comparison. The strongest explanatory variable for each growth form is highlighted by different color. Significance levels of each explanatory variable: *, p < 0.05, **, p < 0.01.

|  | All species | | Herbaceous species | | Shrubs | | | Trees | |
| --- | --- | --- | --- | --- | --- | --- | --- | --- | --- |
|  | Slopes | R^2^  (%) | Slopes | R^2^  (%) | Slopes | R^2^  (%) | Slopes | | R^2^  (%) |
| **Temperature** |  |  |  |  |  |  |  | | |
| MAT | 8.0* | 71.7 | 6.2* | 61.4 | 3.5* | 39.8 | 4.3* | | 41.6 |
| MTCQ | 7.7* | 74.3 | 5.7* | 59.6 | 3.3 | 36.2 | 4.2* | | 46.4 |
| **Precipitation** |  |  |  |  |  |  |  | |  |
| MPWQ | 7.0* | 69.1 | 5.3* | 62.5 | 4.3* | 60.2 | 4.2* | | 46.6 |
| AI | 6.9* | 60.4 | 5.2* | 53.7 | 4.3* | 56.1 | 4.0* | | 39.2 |
| **Seasonality** |  |  |  |  |  |  |  | |  |
| TSN | -5.1 | 29.8 | -3.5 | 19.9 | -1.5 | 7.7 | -3.1* | | 24.2 |
| PSN | -4.6 | 27.5 | -3.1 | 17.5 | -2.2 | 16.1 | -2.0* | | 10.2 |
| **Productivity** |  |  |  |  |  |  |  | |  |
| NPP | 6.7* | 67.5 | 5.2** | 61.6 | 4.0* | 54.2 | 4.3* | | 46.4 |
| GPP | 7.1** | 73.2 | 5.5** | 67.6 | 4.2* | 59.2 | 4.3* | | 47.3 |
| AET | 8.0* | 82.3 | 6.4* | 75.4 | 4.5* | 66.5 | 4.5* | | 51.2 |
| **Functional trait** |  |  |  |  |  |  |  | |  |
| log(Height) | 9.9** | 94.6 | 7.6** | 83.1 | 4.9* | 71.0 | 5.6* | | 54.2 |
| **Evolution** |  |  |  |  |  |  |  | |  |
| Genus Age | 8.2* | 75.9 | 6.5* | 68.1 | 4.6* | 60.0 | 4.7* | | 47.4 |

**Table S5** The best models explaining the proportions of fleshy-fruited species selected by Bayesian model averaging (BMA) method for grid cells with more than 10 species. For each potential variable in the best model, the posterior probability, explanatory power (R^2^), AIC, and p-value of each model were estimated.

| Growth form | Model | | posterior probability | R^2^ (%) | AIC | p-value |
| --- | --- | --- | --- | --- | --- | --- |
| All species | Model 1 | Height | 0.537 | 94.6 | 1260.4 | <0.01 |
|  | Model 2 | Height+Age | 0.255 | 96.5 | 1266.4 | <0.01 |
|  | Model 3 | Height+AET | 0.151 | 96.21 | 1265.8 | <0.01 |
|  | Total | Height+Age+AET | - | 96.76 | 1268.9 | <0.01 |
| Herbaceous species | Model 1 | Height | 0.949 | 83.14 | 382.4 | <0.01 |
|  | Model 2 | Height+AET | 0.051 | 85.56 | 384.7 | <0.01 |
|  | Total | Height+AET | - | 85.56 | 384.7 | <0.01 |
| Shrubs | Model 1 | Height + AET | 0.524 | 75.70 | 3497.5 | <0.01 |
|  | Model 2 | Height + Age | 0.189 | 74.93 | 3522.2 | <0.01 |
|  | Model 3 | Height + MPWQ | 0.152 | 74.76 | 3535.3 | <0.01 |
|  | Total | Height +AET +MPWQ+ Age | - | 76.21 | 3497.4 | <0.01 |
| Trees | Model 1 | Height + Age | 0.351 | 57.06 | 3301.7 | <0.01 |
|  | Model 2 | Height | 0.228 | 55.7 | 3302.3 | <0.01 |
|  | Model 3 | Height + AET | 0.176 | 55.76 | 3302.3 | <0.01 |
|  | Total | Height + AET + Age | - | 57.45 | 3285.3 | <0.01 |

**Table S6** The variance inflation factors (VIF) of variables in best models for each species groups.

|  | **log(Height)** | **AET** | **Age** | **MAT** | **MPWQ** |
| --- | --- | --- | --- | --- | --- |
| **All species** | 3.724 | 5.046 | 3.433 | - | - |
| **Herbaceous species** | 3.692 | 3.692 | - | - | - |
| **Shrubs** | 3.059 | 14.151 | 4.388 | - | 9.561 |
| **Trees** | 6.074 | 6.367 | 4.440 | 3.025 | - |

**Table S7** The spatial autoregressions with spatial error model for the relationships between proportion of fleshy-fruited species and the predictors in the best models selected by Bayesian model averaging (BMA) method. The regression coefficients and R^2^ have been multiplied by 100.

| Growth form | Model | Variable | Regression coefficients | p-value | Lambda | R^2^ (%) | AIC |
| --- | --- | --- | --- | --- | --- | --- | --- |
| All species | ~Height+AET  +Age | Height | 4.10 | <0.01 | -1.14 | 92.9 | -15598 |
|  |  | AET | 4.39 | < 0.01 |  |  |  |
|  |  | Age | 2.76 | < 0.01 |  |  |  |
| Herbaceous species | ~Height+AET | Height | 1.48 | <0.01 | -0.85 | 86.0 | -21878 |
|  |  | AET | 1.92 | <0.01 |  |  |  |
| Shrubs | ~Height+AET+  Age+MPWQ | Height | 6.63 | <0.01 | -0.30 | 80.3 | -11041 |
|  |  | AET | 4.49 | <0.01 |  |  |  |
|  |  | Age | 1.53 | <0.01 |  |  |  |
|  |  | MPWQ | -0.11 | <0.01 |  |  |  |
| Trees | ~AET+Age+ MAT | Height | 2.10 | <0.01 | -0.53 | 62.97 | -7286 |
|  |  | AET | 4.06 | <0.01 |  |  |  |
|  |  | Age | 3.27 | <0.01 |  |  |  |
|  |  | MAT | 3.08 | <0.01 |  |  |  |
