# Supplementary material for: Effects of Climate, Plant Height, and Evolutionary Age on Geographical Patterns of Fruit Type
Source: Front Plant Sci. 2021 Mar 16;12:604272. doi: 10.3389/fpls.2021.604272 (PMC8007967; doi:10.3389/fpls.2021.604272)
Supplement: Supplementary file 2 [file Data_Sheet_2.docx]

**Supporting Information:**

**APPENDIX S2**

**Database: data sources for compiling distribution data**

An,D.G.(2002).*Higher flora of Xiaolong mountain in Gansu.* Gansu national press. Lanzhou. (安定国. (2002).甘肃省小陇山高等植物志, 甘肃民族出版社,兰州)

Ayigamari keram(2015).A Floraistic Analysis on the Medicinal plants in The West Tianshan Mountains.*Xinjiang University.* pp 106.(阿依加马力·克然木. (2015) 西天山药用植物资源区系分析. pp 106. 新疆大学.)

Bai,S.L.& Wu,D.C.(1994).*Flora of Greater Khingan Mountains*. Heilongjiang Science and Technology Press. Haerbin.(柏松林,吴德成.(1994).中国大兴安岭植物志, 黑龙江省科学技术出版社,哈尔滨)

Changbai Mountain Forest Ecosystem Positioning Station, Chinese Academy of Sciences(1982).*The plant list of Changbai mountain.* Changbai Mountain Forest Ecosystem Positioning Station.Yanji. (中国科学院长白山森林生态系统定位站.(1982).长白山植物名录. 中国科学院长白山森林生态系统定位站.延吉.)

Chen,H.B.,Zheng,Y.J., Li,F.Z.(1997).*Flora of Shandong (Vol.1- Vol.2).* Qingdao Press. Qingdao.(陈汉斌,郑亦津,李法曾(1997).山东植物志(上下卷).青岛出版社.青岛)

Chen,S.L.(1962). *Gramineae Flora of east Chian.* Jiangsu Renmin Press. Nanjing. (陈守良.(1962).华东禾本科植物志, 江苏人民出版社,南京)

Chen,X.Q.(2002).*Higher plants of Chine(Vol.13).*Qingdao Press.Qingdao.(陈心启.(2002).中国高等植物第十三卷, 青岛出版社,青岛)

Chen,X.X.,Zhou,Z.,Hu,R.Y.,Ding,B.Y.(2013). Three New Records of Vascular Plants in Zhejiang Province. *Journal of Wenzhou University(Natural Sciences)* .34, 54-56.(陈贤兴, 周庄, 胡仁勇 & 丁炳扬 (2013) 浙江维管植物新纪录. 温州大学学报(自然科学版), 34, 54-56.)

Chen,Y.O. (2015).The Study of Higher Plant Diversity in Dalian Chengshantou Coastal Landform National Nature Reserve. *Liaoning Normal university*.pp 58.(陈雨鸥. (2015) 大连城山头海滨地貌国家级自然保护区高等植物多样性研究., pp 58. 辽宁师范大学.)

Cheng,C.H.(2016). Rare and Endangered Plants in Wudaoxia Nature Reserve and Countermeasures for Protection. *Hubei Forestry Science and Technology*. 45, 69-72.(程传宏. (2016) 五道峡自然保护区珍稀濒危植物及其保护对策. 湖北林业科技, 45, 69-72.)

Cheng,J.Q.,Yang.Z.P.,Liu.P.(1992). *Timber flora of china.* China Forestry Press.Beijing.(成俊卿,杨宗驹,刘鹏. (1992).中国木材志, 中国林业出版社,北京)

China Arboreal Editorial committee (1983). *Tree flora of China.* China Forestry Press.Beijing.(中国树木志编辑委员会.(1983).中国树木志, 中国林业出版社,北京)

Chu.M(1999). *Fruit flora of china(Vol. Plum).* China Forestry Press. Beijing.(褚孟.(1999).中国果树志-梅卷, 中国林业出版社,北京)

Cui,Y.W.(1953). *Essentials of North China Economic Plants.* Science Press. Beijing.(崔友文.(1953).华北经济植物志要, 科学出版社,北京)

Department of Health of Zhejiang Province (1960). *Medicinal flora of Tianmu mountains,Zhengjiang.* Zhengjiang renmin press. Hangzhou.(浙江省卫生厅.(1960).浙江天目山药用植物志, 浙江人民出版社,杭州)

Department of Pharmacy, Shanghai First Hospital(1961). *Medicinal flora of Hangzhou.* Shanghai Science and Technology Press.Shanghai.(上海第一医院药学系生药学教研组.(1961).杭州药用植物志.上海科学技术出版社.上海)

Deng,L.B.,Yan,W.,Wang,G.Q.(2014) The study on the flora of the vascular bundle plants of the Lake-AHA wetland park in Guiyang. *Journal of Guizhou Normal University(Natural Sciences)* .32,6-9.(邓立斌, 颜伟,汪贵庆 (2014) 贵州贵阳阿哈湖国家湿地公园维管植物区系初步研究. 贵州师范大学学报(自然科学版), 32, 6-9.)

Deng.M.Q & Lei,J.J.(2005). *Fruit flora of china(Vol. Strawberry).* China Forestry Press.Beijing.(邓明琴,雷家军.(2005).中国果树志-草莓卷, 中国林业出版社,北京)

Du,Y.B.(2000). Wile resource Flora of Hebei. Hebei University Press.Baoding.(杜怡斌.(2000).河北野生资源植物志, 河北大学出版社,保定)

Economic flora of Anhui Renovation Office(1990). E*conomic flora of Anhui.* Anhui Science and Technology Press. Hefei.(安徽经济植物志增修办公室. (1990).安徽经济植物志. 安徽科学技术出版社,合肥)

Economic flora of Henan editorial committee (1963).  *Economic flora of Henan.* Henan Renmin Press. Zhengzhou.(河南经济植物志编辑委员会.(1963).河南经济植物志, 河南人民出版社,郑州)

Flora of Anhui collaborating group (1986). *Flora of Anhui.* Anhui Science and Technology Press.Hefei.(安徽植物志协作组. (1986).安徽植物志(1-5卷). 安徽科学技术出版社.合肥)

Flora of Guizhou editorial committee (2000). *Flora of Guizhou (Vol.1 - Vol.10).* Guizhou Science and Technology Press. Guiyang.(贵州植物志编辑委员会(1982-2004).贵州植物志(1-10卷).贵州科技出版社.贵阳.)

Flora of Hebei editorial committee (1986). *Flora of Hebei (Vol.1 - Vol.3).* Hebei Science and Technology Press. Shijiazhuang.(河北植物志编辑委员会(1986).河北植物志(1-3卷).河北科学技术出版社.石家庄.)

Flora of Henan editorial committee (2000). *Flora of Henan (Vol.1 - Vol.4).* Henan Science and Technology Press. Zhengzhou. (河南植物志编纂委员会(1993-1997).河南植物志(1-4卷).河南科技出版社.郑州)

Flora of Hunan editorial committee (2000). *Flora of Hunan (Vol.1 - Vol.3).* Hunan Science and Technology Press. Changsha. (湖南植物志编辑委员会.(2000).湖南植物志(1-3卷).湖南科技出版社.长沙.)

Flora of Inner Mongolia editorial committee (1985-1998). *Flora of Inner Mongolia (Vol.1 - Vol.6).* Inner Mongolia Renmin Press. Huhehaote. (内蒙古植物志编辑委员会(1985-1998).内蒙古植物志(1-6卷).内蒙古人民出版社.呼和浩特)

Flora of Jiangxi editorial committee (2014). *Flora of Jiangxi (Vol.1 - Vol.6).* Jiangxi Science and Technology Press.Nanchang.(江西植物志编辑委员会(2014).江西植物志(2-3卷).江西科学技术出版社.南昌)

Flora of Shanxi editorial committee (2004).*Flora of Shanxi (Vol.1- Vol.5).* China Science and Technology Press, Beijing(山西植物志编辑委员会(2004).山西植物志(1-5卷).中国科学技术出版社.北京)

Flora of Sichuan editorial committee (1981). *Flora of Sichuan(Vol.1-Vol.16;Vol.21)* Sichuan Renmin Press, Chengdu.(四川植物志编辑委员会(1981).四川植物志(1-16卷;21卷).四川人民出版社.成都)

Flora of Xinjiang editorial committee (1992-1996). *Flora of Xinjiang(Vol.1 - Vol.6).* Xinjiang science, technology and health press. Wulumuqi.(新疆植物志编辑委员会(1992-1996).新疆植物志(1-6卷).新疆科技卫生出版社.乌鲁木齐)

Flora of Zhejiang editorial committee (1992-1993). *Flora of Zhejiang (Vol.2- Vol.7).* Zhejiang Science and Technology Press. Hangzhou.(浙江植物志编辑委员会(1992-1993).浙江植物志（2-7卷）.浙江科学技术出版社.杭州)

Fu,G.A. & Hong,X.J.(2008). Flora of vascular plants of Jianfengling, Hainan Island. *Guihaia*. 2,226-229.(符国瑷 & 洪小江 (2008) 海南岛尖峰岭的维管植物区系. 广西植物, 226-229.)

Fu,L.G.(2000). *Higher plants of Chine(Vol.3)*.Qingdao Press. Qingdao.(傅立国.(2000).中国高等植物第三卷, 青岛出版社,青岛)

Fu,L.G.(2001). *Higher plants of Chine(Vol.8)*.Qingdao Press. Qingdao.(傅立国.(2001).中国高等植物第八卷, 青岛出版社,青岛)

Fu,L.G.(2003). *Higher plants of Chine(Vol.6)*.Qingdao Press. Qingdao.(傅立国.(2003).中国高等植物第六卷, 青岛出版社,青岛)

Fu,L.G.(2004). *Higher plants of Chine(Vol.10)*.Qingdao Press. Qingdao.(傅立国.(2004).中国高等植物第十卷, 青岛出版社,青岛)

Fu,L.G.&Hong,T.(2005).*Higher plants of Chine(Vol.11)*. Qingdao Press. Qingdao.(傅立国,洪涛.(2005).中国高等植物第十一卷, 青岛出版社,青岛)

Fu,P.Y.(1998). *Herbaceous flora of Northeast China(Vol.1 - Vol.12)*.Science Press. Beijing.(傅沛云(1998).东北草本植物志(1-12卷).科学出版社.北京)

Gansu Beekeeping Research Institute(1987). *Nectariferous flora of Gansu.* Gansu Science and Technology Press. Lanzhou.(甘肃省养蜂研究所(1987).甘肃蜜源植物志, 甘肃科学技术出版社,兰州)

Gao,W.H.(1998).*Flora of Kongtong Mountains.* Gansu cultural Press. Lanzhou.(高维衡.(1998).崆峒山植物志, 甘肃文化出版社,兰州)

Ge,B.F.,Zhang,C.C.,Su,F.,Zhang,J.C.(2011). List of wild plants in Ji'an. *Chinese Horticulture Abstracts.* 27, 51-56.

葛宝福, 张绪成, 苏峰 & 张金刚 (2011) 集安市山野菜植物名录. 中国园艺文摘, 27, 51-56.

Guangxi institute of botany, Chinese academy of sciences (1991). *Flora of Guangxi (Vol.1 - Vol.6)*. Guangxi Science and Technology Press. Nanning.(中国科学院广西植物研究所(1991).广西植物志(1-6卷). 广西科学技术出版社.南宁.)

Guo,S.J.(1993). *Fruit flora of china (Vol. Ginkgo).* China Forestry Press. Beijing.(郭善基.(1993).中国果树志-银杏卷, 中国林业出版社,北京)

Hao,X.L.(2014) The analyse of Floristic Geography and Vegetation types of tracheophytes in the extremely arid desert National Nature Reserve of Anxi，Gansu. *Lanzhou University.* pp 178.(郝小玲. (2014) 甘肃安西极旱荒漠国家级自然保护区维管植物地理区系与植被类型分析., pp 178. 兰州大学.)

He,G.S.(2008). New distribution records of eight vascular plants in Fujian Province. *Journal of Fujian Forestry Science and Technology.* 93-94&106.(何国生 (2008) 福建8种维管束植物分布新记录. 福建林业科技, 93-94&106.)

He,S.Y.,et al.(1993).*Flora of Beijing.* Beijing Press. Beijing.(贺士元等(1993).北京植物志(上下册).北京出版社,北京)

Hu,J.,Xiong,Y.N.,Wu,X.G.,Chen,Q.H.,Liu.Q.(2016). New Records of Vascular Plants in Xizang .*Acta Botanica Boreali-Occidentalia Sinica.* 36, 2332-2338.(胡君, 熊豫宁, 伍小刚, 陈庆恒 & 刘庆 (2016).西藏维管植物新记录. 西北植物学报, 36, 2332-2338.)

Huang,D.Z.,Bai,T,R.(1964). *Economic Timber Flora of Northeast China*. Science Press. Beijing.(黄达章,白同仁.(1964).东北经济木材志. 科学出版社,北京)

Huang.J.X.,Li,X.,Qian,J.Y.(1996).*Flora of Saihanba*. China Science and Technology Press.Bejing.(黄金祥,李信,钱进源.(1996).塞罕坝植物志, 中国科学技术出版社,北京)

Huang,X.W.,Liang,X.M.,Wu,L.J.,Zhang,X.M.(2009). Floristic Analysis of Vascular Plants in Dalai Lake National Nature Reserve of Inner Mongolia. J*ournal of Anhui Agricultural Sciences*. 37, 3131-3133 & 3185. (黄学文, 梁秀梅, 乌力吉 & 张晓明 (2009) 内蒙古达赉湖自然保护区维管植物科属区系分析. 安徽农业科学, 37, 3131-3133+3185.)

Huang,Y.S.(2010).Study on Species Diversity of Plants in The Plot of Important Forest Species Resources in Longgang Natural Reserve. *Guangxi Normal University.* pp 155.(黄俞淞. (2010) 弄岗自然保护区重要森林物种资源监测样地植物物种多样性研究., pp 155. 广西师范大学.)

Institute of Agricultural Biology, Sichuan Branch, Chinese Academy of Sciences (1962-1963). *Wild economic flora of Sichuan.* Sichuan Renmin Press. Chengdu.(中国科学院四川分院农业生物研究所.(1962-1963).四川野生经济植物志, 四川人民出版社,成都)

Institute of Botany, Chinese Academy of Sciences(1974). *Flora of Qin Mountains(Vol.1)* .Science Press. Beijing.(中国科学院植物研究所.(1974).秦岭植物志(第一卷).科学出版社.北京)

Institute of Botany,Jiangsu Province and Chinese academy of sciences(2017). *Flora of Jiangsu (Vol.2- Vol.5).* Jiangsu Renmin Press. Nanjing.(江苏省植物研究所(2017).江苏植物志(2-5卷).江苏人民出版社.南京)

Institute of Forestry and Soil, Chinese Academy of Sciences(1960). *Economic flora of Liaoning.* Liaoning Renmin Press. Shenyang.(中国科学院林业土壤研究所 (1960).辽宁经济植物志.辽宁人民出版社.沈阳)

Institute of Forestry and Soil, Chinese Academy of Sciences (1959). *Medicinal flora of Northeast China.* Science Press. Beijing.(中国科学院林业土壤研究所.(1959).东北药用植物志.科学出版社.北京)

Institute of Forestry and Soil, Chinese Academy of Sciences (1959). *Illustrated woody flora of Northeast China.* Science Press. Beijing.(中国科学院林业土壤研究所.(1955).东北木本植物图志.科学出版社.北京)

Ji.X.L.(2007). Studies on plant Diversity Conservation of Forest Community in Yaoluoping National Nature Reserve. *Anhui Agricultural University.* pp 62.(吉兴磊. (2007) 鹞落坪国家级自然保护区森林群落植物多样性保护研究., pp 62. 安徽农业大学.)

Ji,T.(2007). Study on Species Diversity and Ecological Planning of Hongze Lake Wetland National Nature Reserve. *Nanjing Forestry University*. pp 79.(纪涛. (2007) 洪泽湖湿地国家级自然保护区物种多样性与生态规划研究., pp 79. 南京林业大学.)

Jia,J.X., Jia,D.X.,Ren,Q.M.(2006). *Chinese crops and their wild relatives-fruit tree volume.* China Agriculture Press.Beijing.(贾敬贤,贾定贤,任庆棉.(2006).中国作物及其野生近缘植物-果树卷.中国农业出版社,北京)

Jiangsu Provincial Department of Commerce & Institute of Botany,Jiangsu Province and Chinese academy of sciences(1959).*Wild flora of Jiangsu.* Jiangsu Renmin Press. Nanjing.(江苏省商业厅,中国科学院植物研究所南京中山植物园编 (1959).江苏野生植物志, 江苏人民出版社,南京)

Jin,S.(2009). Study on plant diversity and its conservation in Ningxia Helan Mountain National Nature Reserve.*Beijing Forestry University*. pp 163.(金山. (2009) 宁夏贺兰山国家级自然保护区植物多样性及其保护研究., pp 163. 北京林业大学.)

Kunming institute of botany, Chinese academy of sciences. *Flora of Xizang(Vol.1 - Vol.5)*. Science Press. Beijing. (中国科学院昆明植物研究所(1983).西藏植物志(1-5卷).科学出版社.北京)

Kunming institute of botany, Chinese academy of sciences. *Flora of Yunnan(Vol.1 - Vol.16)*. Science Press. Beijing. (中国科学院昆明植物研究所(1997-2006). 云南植物志(1-16卷).科学出版社.北京)

Lanzhou Institute of Desert Research, Chinese Academy of Sciences(1992).*Flora of desert area in China.* Science Press. Beijing.(中国科学院兰州沙漠研究所. (1992).中国沙漠植物志, 科学出版社,北京)

Le,T.Y.,Xu,W.Y.(1957).*Flora of basin in Shannxi,Gansu,and Ningxia.* China Forestry Press.Beijing.(乐天宇,徐纬英.(1957).陕甘宁盆地植物志, 中国林业出版社,北京)

Li,C.G.,Wang,Y.M.,Xuan,W.(1990). *Wild economic flora of Nontheast area of Changbai Mountains.*Yanbian Renmin Press.Yanbian.(李春光,王永明,玄武.(1990).长白山东北部野生经济植物志, 延边人民出版社,延边)

Li,C.R.,Deng,L.X.,Li,M.,He,W.(2013). Investigation on the Endangered and Rare Plant Resource in Xishui National Nature Reserve. *Guizhou Forestry Science and Technology*. 40, 1-7.(李从瑞, 邓伦秀, 李茂 & 何伟 (2012) 贵州习水国家级自然保护区珍稀濒危维管束植物资源调查. 贵州林业科技, 40, 1-7.)

Li,D.W.(2008) The FLORISTIC GEOGRAPHY OF VASCULAR PLANTS OF THE LOESS PLATEAU OF NORTHERN SHAANXI PROVINCE. *Northwest Agricultural and Forestry University.* pp 201.(李登武. (2008) 陕北黄土高原维管植物区系地理研究., pp 201. 西北农林科技大学.)

Li,F.Z.,Li,W.Q.,Fan,S.J.(2016).*Woody flora of Shandong(Vol.1,Vol.2)*. Science Press. Beijing.(李法曾,李文清,樊守金.(2016).山东木本植物志(上下卷). 科学出版社.北京)

Li,F.Z.,Zhao,Z.T.(2004). *Essentials of Shandong Plants.* Science Press. Beijing.(李法曾,赵遵田.(2004).山东植物精要. 科学出版社,北京)

Li,H.M.(1990). *Economic flora of Shanxi.* China Forestry Press.Beijing.(李惠民.(1990).山西省经济植物志, 中国林业出版社,北京)

Li,L.P.(2005). Vegetation Classification and Evaluation of Key Protected Plants in Beijing Wuling Mountain Nature Reserve. *Beijing Forestry University.* pp 103.(李利平. (2005) 北京雾灵山自然保护区植被分类与重点保护植物评价., pp 103. 北京林业大学.)

Li,R.M.,ShangGuan,T.L.,Zhang,J.L.,Hao,J.(2015). H*engshan Resources Plant History (Vol.1-Vol.2).* Shanxi Science and Technology Press.Taiyuan.(李日明,上官铁梁,张吉林,郝婧.(2015).恒山资源植物志要(上下册).山西科技出版社.太原)

Li,S.X. (1988-1992). *Flora of Liaoning (Vol.1- Vol.2).* Liaoning Science and Technology Press. Shenyang.(李书心(1988-1992).辽宁植物志(上下卷).辽宁科学技术出版社.沈阳)

Li,Y.J.(1987).*Woody flora of Qinghai.* Qinhai Renmin Press.Xining.(李耀阶.(1987).青海木本植物志, 青海人民出版社,西宁)

Liang,G.L. & Yi,S.R.(2013). *Resources of wild medicinal plants in Jinfoshan mountains.* China Science and Technology Press.Bejing.(梁国鲁,易思荣.(2013).金佛山野生药用植物资源.中国科学技术出版社.北京)

Liao,J.H.,Dai,L.Y.,He,W.Q.(2007). Investigation and protective utilization of vascular plant resources in Luofu Mountain. *Journal of Hunan Agricultural University(Natural Sciences)*.3,281-284.(廖建良 & 戴良英 & 贺握权 (2007) 罗浮山维管植物资源调查及保护利用. 湖南农业大学学报(自然科学版), 3,281-284.)

Lin,L.G.,Lin,Y.Y.,Zhang,Y.T.(1981). List of Vascular Plants in Wuyishan Nature Reserve. *Wuyi Science Journal.* 1, 17-69.(林来官 & 林有润 & 张永田 (1981) 武夷山自然保护区维管束植物名录. 武夷科学, 1, 17-69.)

Liu,J.Y.(2004). *Flora of Tianjin.* Tianjin Science and Technology Press.Tianjin.(刘家宜(2004).天津植物志.天津科学技术出版社,天津)

Liu,B.B.(2013). Geographical Study on the Seed Plant Flora in the Northern Section of Donggong Mountain. Zhejiang *Agricultural and Forestry University.* pp 181.(刘彬彬. (2013) 洞宫山北段种子植物区系地理研究., pp 181. 浙江农林大学.)

Liu,D.T.,Li,W.S.,He,Y.L.,Chen,Z.F.,Huang,H.,Gao,F.,Xu,K.,Wu,Z.K.(2015). Resources Evaluation of Seed Plants in Yulong Snow Mountain, Lijiang, Northwestern Yunnan. *Plant Diversity and Resources.* 37, 318-326.(刘德团, 李婉莎, 和玉龙, 陈智发, 黄华, 高富, 许琨 & 吴之坤 (2015) 玉龙雪山种子植物资源评价. 植物分类与资源学报, 37, 318-326.)

Liu,J.J.,Wang,L.M.,Miao,Q.,Wang,Y.J.(2009). Study on Resource Plants and Diversity of Taikuanhe Nature Reserve, Shanxi. *Journal of Shanxi University (Natural Science Edition).* 32, 477-482.(刘晶晶, 王良民, 苗青 & 王钰婧 (2009) 山西太宽河自然保护区资源植物研究. 山西大学学报（自然科学版）, 32, 477-482.)

Liu,L.K.,Yuan,Z.K.,Lin,B.,Wen,S.L.,She,J.M.(2008). Investigation of Vascular Plant Resources in Yingzuijie National Nature Reserve, Hunan. *Journal of Southwest China Normal University (Natural Science Edition).* 4,53-57.(刘良科, 袁正科, 林柏, 文声陆 & 佘晶明 (2008) 湖南鹰嘴界国家级自然保护区维管植物资源调查. 西南师范大学学报(自然科学版), 4,53-57.)

Liu,M.J.(1998).*Wild fruit tree in China.* Agriculture Press.Beijing.(刘孟军.(1998).中国野生果树, 农业出版社,北京)

Liu,N.Y.(1993). *Revitalized general annals of Taiwan Province(Vol.2)*.Taiwan Provincial Documentation Committee:plant chapter.Nantou,Taiwan.(刘宁颜.(1993).重修台湾省通志卷二-土地志-博物篇-植物章, 台湾省文献委员会,南投市,台湾省)

Liu,Q.R.& Kang,M.Y.(2008). Some Newly Recorded Vascular Plants from Shanxi. *Acta Botanica Boreali-Occidentalia Sinica.* 4,2412-2415.(刘全儒 & 康慕谊 (2008) 山西维管植物新资料. 西北植物学报, 4,2412-2415.)

Liu,T.W.& Zhang,Y.F.(1990). *Flora of Taiyuan.* China Science and Technology Press.Bejing.(刘天慰,张云峰. (1990).太原植物志.中国科学技术出版社.北京)

Liu,X.L.,Yin,L.K.,Liu.B.,Niu,S.H.,Yang,H.(2016). Tien Shan Tomur Grand Canyon Wild Seed Plant List. *Journal of Green Science and Technology.*13,8-9.(刘旭丽, 尹林克, 刘彬, 牛淑华 & 杨寒 (2016) 天山托木尔大峡谷野生种子植物名录. 绿色科技,13,8-9.)

Long,M.(2013). Study on the flora and plant resources of Helan Mountain National Nature Reserve in Inner Mongolia. *Inner Mongolia Normal University.* pp 122.(龙梅. (2012) 内蒙古贺兰山国家级自然保护区植物区系及其植物资源研究., pp 122. 内蒙古师范大学.)

Lu,J.L.,Yu,X.Y.,Zhang,J.P.(1998). *Woody plants illustrated book in Henan.* New century Press.Guangzhou.(卢炯林 余学友 张俊朴.(1998).河南木本植物图鉴, 新世纪出版社,广州)

Lu,P.P.(2010). Study on Plant Diversity and Protection in Hanma National Nature Reserve. Northeast Forestry University. pp 92.(卢平平. (2010) 汗玛国家级自然保护区植物多样性及其保护研究., pp 92. 东北林业大学.)

Lu,Q.,Wang,J.H.,Chu,J,M.(2012). *Illustrated flora of Chinese desert.* China Forestry Press.Beijing.(卢琦,王继和,褚建民.(2012). 中国荒漠植物图鉴.中国林业出版社,北京)

Lu,Q.N.& Jia,D.X.(1999). *Fruit flora of china(Vol. Apple).* China Forestry Press.Beijing.(陆秋农,贾定贤. (1999).中国果树志-苹果卷, 中国林业出版社,北京)

Lu,Y.H. (2012a) List of wild plants of Guangxi very small population(1). *Forestry of Guangxi.*6, 47.(卢燕华(2012a) 广西极小种群野生植物名录(上). 广西林业, 47.)

Lu,Y.H. (2012b) List of wild plants of Guangxi very small population(2). *Forestry of Guangxi.*6, 47.(卢燕华 (2012b) 广西极小种群野生植物名录(下). 广西林业, 47.)

Luo,K.W.(2016). Resources of vascular plants in Guangxi Wanggangshan Nature Reserve. *Hunan Forestry Science & Technology.* 43, 93-97.(罗开文 (2016) 广西王岗山自然保护区维管植物资源. 湖南林业科技, 43, 93-97.)

Luo,W. (2015). The study on plant flora and the characteristics of plants community of Nanhaizi Wetland Nature Reserve. *Inner Mongolia Agricultural University.* pp 94. (罗伟. (2015) 南海子湿地自然保护区植物区系与植物群落特征研究., pp 94. 内蒙古农业大学.)

Ma,D.Z.(2007). *Flora of Ningxia (Vol.1- Vol.2).* Ningxia Renmin Press. Yinchuan.(马德滋(2007).宁夏植物志(上下册).宁夏人民出版社.银川)

Mao,Z.Z.(1997). Investigation of Guangxi Plant List. *Guihaia.* 2,92-97.(毛宗铮 (1997). 广西植物名录考. 广西植物, 92-97.)

Mei,Z.F.(1999). *Common trees in Beijing.* China Forestry Press. Beijing. (梅志奋.(1999).北京常见树木, 中国林业出版社,北京)

Nansha Comprehensive Scientific Investigation Team of the Chinese Academy of Sciences(1996).*Flora of Spratly Islands and its neighboring islands.* Ocean Press.Beijing.(中国科学院南沙综合科学考察队.(1996).南沙群岛及其邻近岛屿植物志, 海洋出版社,北京)

Niu,C.S.(1990).*Tree flora of Shannxi.* China Forestry Press.Beijing.(牛春山.(1990).陕西树木志, 中国林业出版社,北京)

Northwest botanical institute, Chinese academy of sciences(2000).*Flora of Loess Plateau.* Science Press. Beijing.(西北植物研究所编著.(2000).黄土高原植物志, 科学出版社,北京)

Northwest Institute of Plateau Biology, Chinese academy of sciences (1996). *Flora of Qinghai (Vol.1- Vol.4).* Qinghai Renmin Press. Xining. (中国科学院西北高原生物研究所(1996).青海植物志.青海人民出版社.西宁)

Office of Agricultural Division Committee of Tonghua District, Jilin Province.(1985).*Wild economic flora of Southwest slope of Changbai Mountains.* Office of Agricultural Division Committee of Tonghua District, Jilin Province.Tonghua.(吉林省通化地区农业区划委员会办公室.(1985).长白山西南坡野生经济植物志.吉林省通化地区农业区划委员会办公室,通化)

Office of Agricultural Division Committee of Tonghua District, Jilin Province.(1961). *Wild economic flora of Jilin.* Jilin Renmin Press.(吉林省通化地区农业区划委员会办公室.(1961).吉林省野生经济植物志. 吉林人民出版社,长春)

Peng,R.C. (2013). Vascular bundle flora of Mulun National Nature Reserve in Guangxi.Guangxi Normal University. pp 256.(彭日成. (2013) 广西木论国家级自然保护区维管束植物区系研究., pp 256. 广西师范大学.)

Qi,C.J. & Lin,Q.Z.(2000).*Tree flora of Hunan.* Hunan Science and Technology Press.Changsha.(祁承经,林亲众. (2000).湖南树木志, 湖南科学技术出版社,长沙)

Qi,C.J. & Yu,X.L.(2002). *Overview of seed plants in Hunan.* Hunan Science and Technology Press.Changsha.(祁承经,喻勋林. (2002).湖南种子植物总览, 湖南科学技术出版社,长沙)

Qin,S.,Ge,H.,Zhao,L.Q.(2014). New Data of Vascular Plants in Inner Mongolia. *Acta Botanica Boreali-Occidentalia Sinica.* 34, 397-400.(秦帅 & 葛欢 & 赵利清 (2014) 内蒙古维管植物新资料. 西北植物学报, 34, 397-400.)

Qin,S.,Chen,L,Zang,C.X.,Zhao,L.Q.(2016). New Records of Vascular Plants in the Helan Mountain,Ningxia and Inner Mongolia. *Arid Zone Research*.33, 789-791.(秦帅, 陈龙, 臧春鑫 & 赵利清 (2016) 贺兰山及内蒙古、宁夏维管植物新记录. 干旱区研究, 33, 789-791.)

Qinghai-Tibet Plateau Comprehensive Scientific Investigation Team of Chinese Academy of Sciences(1994)*. Vascular plants in Hengduan Mountains.* Science Press. Beijing.(中国科学院青藏高原综合科学考察队.(1994).横断山区维管束植物, 科学出版社,北京)

Qin,W.H.,Jiang,S.K.,Xu,W.G.,He,Z.H.(2012). Assessment of in situ conservation of 1, 334 native orchids in China. *Biodiversity Science.* 20, 177-183.(秦卫华, 蒋明康, 徐网谷 & 贺昭和 (2012) 中国1,334种兰科植物就地保护状况评价. 生物多样性, 20, 177-183.)

Qiu,W.L. & Zhang,H.Z.(1996) *Fruit flora of china(Vol. Longan-loquat).* China Forestry Press.Beijing.(邱武陵,章恢志. (1996).中国果树志-龙眼-枇杷卷, 中国林业出版社,北京)

Qu,Z.Z. & Wang,Y.H.(1993). *Fruit flora of china(Vol. jujube).* China Forestry Press.Beijing.(曲泽洲,王永蕙. (1993).中国果树志-枣卷, 中国林业出版社,北京)

Qu,Z.Z(1990).*Fruit tree flora of Beijing.* Beijing Press. Beijing.(曲泽洲 (1990).北京果树志, 北京出版社,北京)

Ren,Z.L.(2010) STUDY ON THE FLORA OF VASCULAR PLANTA IN THE JIUFENG MOUNTAIN. *Inner Mongolia University.* pp 72.(任志龙. (2010) 九峰山自然保护区维管植物区系研究., pp 72. 内蒙古大学.)

Shanxi Agricultural Division Commission (1990). *Economic flora of Shanxi.* China Forestry Press.Beijing.(山西省农业区划委员会.(1990).山西省经济植物志, 中国林业出版社,北京)

Shanxi Academy of Forestry(2001).*Woody flora of Shanxi.* China Forestry Press.Beijing.(山西省林业科学研究院.(2001).山西树木志, 中国林业出版社,北京)

Shanxi Agricultural Division Commission (1991). *Illustrated woody flora of Shanxi.* Science Press. Beijing.(山西省农业区划委员会.(1991).山西树木图志, 科学出版社,北京)

Shanghai Academy of Science and Technology(1999). *Flora of Shanghai (Vol.1- Vol.2).* Shanghai Science and Technological Literature Press. Shanghai(上海科学院(1999).上海植物志(上下卷).上海科学技术文献出版社.上海)

Shi,C,K.(2008) The systematic classification and floristic geography of orchids in Gansu province. *Gansu Agricultural University.* pp 153.(石昌魁. (2008) 甘肃省兰科植物系统分类与区系地理., pp 153. 甘肃农业大学.)

Shu,Z.F.,Lyu,J.,Song,X.J.,Huo,Z.M.,Zhang,C.,Yang,L.,Xiao,R.G.,Luan,X.L.(2017). Statistic of the Vascular Plant Specimens from Chebaling National Nature Reserve in Guangdong Provin. *Forestry and Environmental Science.* 33, 61-65.(束祖飞, 吕江, 宋相金, 霍兆敏, 张超, 陈敏豪, 杨立, 肖荣高 & 栾晓峰 (2017) 广东车八岭国家级自然保护区维管植物标本的整理与分析. 林业与环境科学, 33, 61-65.)

South China Botanical Garden, Chinese academy of sciences (1964-1977). *Flora of Hainan (Vol.1 - Vol.4).* Science Press. Beijing.(中国科学院华南植物研究所(1964-1977).海南植物志(1-4卷).科学出版社.北京)

South China Botanical Garden, Chinese academy of sciences(1956).*Flora of Guangzhou.* Science Press. Beijing.(中国科学院华南植物研究所. (1956). 广州植物志, 科学出版社,北京)

South China Botanical Garden, Chinese academy of sciences (1987). *Flora of Guangdong.*Guangdong Science and Technology Press. Guangzhou.(中国科学院华南植物研究所. (1987).广东植物志(1-9卷). 广东科技出版社,广州)

Southwest Forestry University (1990). *Illustrated woody flora of Yunnan.* Yunnan Science and Technology Press. Kunming.(西南林学院.(1990).云南树木图志,云南科技出版社,昆明)

Sun,H.& Zhou,Z.K.(2002). *Seed plants in the Yarlung Zangbo River Gorge Valley.* Yunnan Science and Technology Press.Kunming.(孙航,周浙昆.(2002).雅鲁藏布江大峡弯河谷地区种子植物,云南科技出版社,昆明)

Sun,X.W.(1962).*General flora of Lanzhou.*Gansu Renmin Press.Lanzhou.(孙宪武.(1962).兰州植物通志, 甘肃人民出版社,兰州)

Tan,P.X.(1983). *Rhododendron Flora of southern China.* Guangdong science press. Guangzhou.(谭沛祥.(1983).华南杜鹃花志, 广东科学出版社,广州)

Tang,C.M.& Pan,X.X.(2012).National Key Protected Wild Plants and Protection Measures in Guangxi Gui － northeast Distict. *Forest Investigation Design.* 3,88-92.(唐初明 & 潘锡样 (2012) 广西桂东北地区国家重点保护野生植物(第一批)及保护措施. 林业勘查设计, 3,88-92.)

Tang,L.H.,Wang,Z.L.,Zhang,F.,Li,G.L.,Yang,Y.X.,He,W.,Zhang,S.L.(2008). Study on the Flora of Vascular Bundle Plants in Saihanwula Nature Reserve. Journal of Anhui Agricultural Sciences.7, 2857-2862.(唐立红, 王志玲, 张帆, 李桂林, 杨永昕, 贺伟 & 张书理 (2008) 赛罕乌拉自然保护区维管束植物区系. 安徽农业科学, 7,2857-2862.)

Tang,Z.F,(2014). Investigations on Vascular Plants Resources in Nulurhushan Nature Reserve in Liaoning Province. Protection Forest Science and Technology.4, 54-56.(汤志馥 (2014) 辽宁努鲁儿虎山自然保护区维管植物资源调查初报. 防护林科技,4,54-56.)

Teng,Y.F.(2013).Study on Plant Diversity of Shahu Nature Reserve in Ningxia*.Ningxia University.* pp 74.(滕迎凤. (2013) 宁夏沙湖自然保护区植物多样性研究., pp 74. 宁夏大学.)

Tian,H.M.(2009) Analysis of seed plant resources and flora characteristics of Huangbaiyu Nature Reserve in Shaanxi Province. *Northwest Agricultural and Forestry University.* pp 50.(田华民. (2009) 陕西省黄柏塬自然保护区种子植物资源与区系特征分析., pp 50. 西北农林科技大学.)

Tian,J.P.,Hu,Y.Y.,Zhang,J.Q.,Liu,M.S.(2008).Survey of resources of vascular medicinal plants endemic in Hainan province. Journal of Hainan Medical College.2, 122-125&128.(田建平, 胡远艳, 张俊清 & 刘明生 (2008) 海南特有维管植物的药用资源. 海南医学院学报, 2,122-125+128.)

*Tree flora of South China.* China Forestry Press.Beijing.(华北树木志编写组. (1984).华北树木志, 中国林业出版社,北京)

Tree flora of Zhongtiao Mountains Editorial committee(1995). *Tree flora of Zhongtiao Mountains*. China Forestry Press.Beijing.(中条山树木志编委会.(1995).中条山树木志, 中国林业出版社,北京)

Tu,W.G.,Gao,X.F.,Liu,S.H.,Wu,N.(2008).Floristics of Vascular Plants on the West Slope of Mt. Jiuding in Wenchuan, Sichuan, China. *Chinese Journal of Applied & Environmental Biology.*3,298-302.(涂卫国, 高信芬, 刘士华 & 吴宁 (2008) 九顶山西坡汶川段维管植物区系研究. 应用与环境生物学报,3,298-302.)

Wang,A.Y.,He,C.X.,Yang,J,H.(2011). The List of Halophytes in Kuitun Area. *Journal of Yili Normal University(Natural Science Edition)*. 2,34-38&54.(王爱英 & 何春霞 & 杨金红 (2011) 新疆奎屯地区野生植物资源调查. 伊犁师范学院学报(自然科学版), 34-38+54.)

Wang,H.,Zahng,C.Q.,Li,D.Z.(2007). *Seed Plant List of Lijiang Alpine Botanical Garden.* Yunnan Science and Technology Press.Kunming.(王红,张长芹,李德铢等.(2007).丽江高山植物园种子植物名录.云南科技出版社.昆明)

Wang,J.J.(2004). Study on Plant Diversity and 3S-Aided Analysis Techniques in Saihanba Nature Reserve.*Beijing Forestry University*.pp 101.(王建军. (2004) 塞罕坝自然保护区植物多样性及3S辅助分析技术研究., pp 101. 北京林业大学.)

Wang,J.Y.,Mu,Q.A.,Lin,R.T.(2008a). Vascular Systematic Checklist of Ecological Corridor in Southern Region of GaoLiGong Nature Reserve(1). Journal of Yunnan Normal University(Natural Sciences Edition). 3,57-63.(汪建云 & 母其爱 & 蔺如涛 (2008a) 高黎贡山自然保护区南段生物走廊带维管植物名录(1). 云南师范大学学报(自然科学版), 3,57-63.)

Wang,J.Y.,Mu,Q.A.,Lin,R.T.(2008b). Vascular Systematic Checklist of Ecological Corridor in Southern Region of GaoLiGong Nature Reserve(2). Journal of Yunnan Normal University(Natural Sciences Edition). 3,52-59.(汪建云 & 母其爱 & 蔺如涛 (2008b) 高黎贡山自然保护区南段生物走廊带维管植物名录(2). 云南师范大学学报(自然科学版), 3,52-59.)

Wang,L.,Shi,S.,Liao,W.B.,Chen,C.Q.,Li,Z.(2013). Rare and endangered plants in Mount Jinggangshan region. *Biodiversity Science.* 21, 163-177.(王蕾, 施诗, 廖文波, 陈春泉 & 李贞 (2013).井冈山地区珍稀濒危植物及其生存状况. 生物多样性, 21, 163-177.)

Wang,Q.,Deng,M.B.,Yang,R.T.,Yao,G.(2011). New records of vascular plants in Jiangsu Province(IV). *Journal of Plant Resources and Environment.*20, 90-91.(汪庆, 邓懋彬, 杨如同 & 姚淦 (2011) 江苏省维管植物分布新记录(四). 植物资源与环境学报, 20, 90-91.)

Wang,S.L.,Luo,J.,Lang,X.D.,Su,J.R.,Zhang,W.Y.(2013). Evaluation of Conservation Priority on Rare and Endangered Plants in Shegyla Mountains, Tibet. *Acta Botanica Boreali-Occidentalia Sinica.* 33, 177-182.(汪书丽, 罗建, 郎学东, 苏建荣 & 张炜银 (2013) 色季拉山珍稀濒危植物优先保护研究. 西北植物学报, 33, 177-182.)

Wang,T.G.,Xing,S.H.,Lin,D.Y.,Wang,J.,Xiao,Y.Q.,Zhao,T.J.,Ren,X.B.(2007). Floristic analysis of vascular bundle plant in Baxianshan Nature Reserve, Tianjin. *Hebei Journal of Forestry and Orchard Research.*2, 134-139.(王天罡, 邢韶华, 林大影, 王娟, 肖雁青, 赵铁建 & 任秀柏 (2007) 天津八仙山自然保护区维管束植物分析. 河北林果研究, 134-139.)

Wang,X.G.,Wu,A.P.,Kang,Z.G.,Gao,B.Q.(1999).*Flora of Heichashan Forest District, Shanxi.* Shanxi Science and Technology Press.Taiyuan.(王喜贵,吴埃平,康志刚,董发盛,高宝琴.(1999).山西黑茶山林区植物志, 山西科学技术出版社,太原)

Wang,Z.H. & Zhuang,E,J.(2001). *Fruit flora of china(Vol. Pink)*. China Forestry Press.Beijing.(汪祖华,庄恩及. (2001).中国果树志-桃卷, 中国林业出版社,北京)

Woody flora of Anhui editorial group (1983). *Woody flora of Anhui.* Anhui Science and Technology Press. Hefei.(安徽木本植物编写组. (1983).安徽木本植物, 安徽科学技术出版社,合肥)

Wuhan Botanical Garden, Chinese academy of sciences(2000). *Flora of Hubei (Vol.1 - Vol.4).* Hubei Science and Technology Press. Wuhan.(中国科学院武汉植物研究所(2002).湖北植物志(1-4卷).湖北科技出版社.武汉)

Wu,L.(2012). Studies on Species Diversity of Plants in Damingshan National Nature Reserve of Guangxi*.Guangxi Normal University.* pp 417.(吴磊. (2012) 广西大明山国家级自然保护区植物物种多样性研究.广西师范大学.)

Wu,S.X.(1998). *Fruit flora of china(Vol. litchi).* China Forestry Press.Beijing.(吴淑娴主编.(1998).中国果树志-荔枝卷, 中国林业出版社,北京)

Wu,Y.H. & Fang.R.Z.(2013). *Flora of Kunlun Mountains.* Chongqing press. Chongqing.(吴玉虎,方瑞征.(2013).昆仑植物志.重庆出版社.重庆.)

Xi,R.T.& Zhang,Y.P.(1996). *Fruit flora of china(Vol. Walnut).* China Forestry Press.Beijing.(郗荣庭,张毅萍.(1996).中国果树志-核桃卷, 中国林业出版社,北京)

Xiang,H.,Zuo,J.H.,Lin,C.S.,Sun,A.Q.,Wang,X.Y.,Liao,W.(2010). Investigation of Medical Vascular Plant Resources in Liuzhi Special Zone of Guizhou Province. *Guizhou Agricultural Sciences.* 38, 19-23.(向红, 左经会, 林长松, 孙爱群, 王绪英 & 廖雯 (2010) 贵州省六枝特区药用维管植物资源调查. 贵州农业科学, 38, 19-23.)

Xiao,Z.X.,Zhan,C.A.,Peng,J.H.,Xie,S.H.,Chen,Y.H.(2010). Investigation and Analysis on Vascular Plant Resources of Reservoirs Area in Shantou. *Guangdong Forestry Science and Technology.* 26, 45-50.(肖泽鑫, 詹潮安, 彭剑华, 谢少鸿 & 陈远合 (2010) 汕头市水库库区维管植物资源调查分析. 广东林业科技, 26, 45-50.)

Xie,H.& Sun,C.(2014) *Guizhou Maple Resource and Utilization Status.* Science Press. Beijing.(谢华,孙超.(2014).贵州桑科植物资源图鉴及利用现状.科学出版社.北京)

Xie,Y.J.(2012). Study on Floristics of vascular plants and plant resources of Guangxi Beibu Gulf Coastal Zone. *Guangxi Normal University.* pp 217.(谢彦军. (2012).广西北部湾海岸带维管植物区系地理与植物资源研究., pp 217. 广西师范大学.)Xin,Z.H.(2002). *Flora of Jiaozuo.* Xian Map Press.Xian.(辛泽华. (2002).焦作植物志, 西安地图出版社.西安.)

Xinjiang Institute of Biological Soil and Desert, Chinese Academy of Sciences(1977)*.Medical flora of Xinjiang.* Xinjiang Renmin Press.Wulimuqi.(中国科学院新疆生物土壤沙漠研究所.(1977).新疆药用植物志, 新疆人民出版社,乌鲁木齐)

Xing,F.W. & Yu,M.S.(2000). *Wild plants in Shenzhen.* China Forestry Press. Beijing.(邢福武,余明思 (2000).深圳野生植物, 中国林业出版社,北京)

Xu,D.Y.(2006). Study on the types, structure and species diversity of restoration communities in the 16-year wind disaster area of Jinyun Mountain Nature Reserve. *Southwest University.* pp 74.(许冬焱. (2006) 缙云山自然保护区16年风灾迹地恢复群落类型、结构及物种多样性特征研究., pp 74. 西南大学.)

Xu,G.L.(2014) .The New Records of the Vascular Plants from Jiulianshan Nature Reserve, Jiangxi Province. *Subtropical Plant Science.* 43, 127-132.(徐国良 (2014) 江西省及九连山地区维管植物新记录. 亚热带植物科学, 43, 127-132.)

Xu,J.M.(2014). INEVSTIGATION AND EVALUATION OF WILD ORNAMENTAL PLANTS RESOURCES IN FANGCHENG YELLOW CAMELLIA NATIONAL NATURAL RESERVE IN GUANGXI.*Guang xi University.* pp 115.(徐竟甯. (2014) 广西防城金花茶国家级自然保护区野生观赏植物资源调查与评价., pp 115. 广西大学.)

Xu,W.B.,Pan,B.,Liang,Y.J.,Zhu,Y.X.,Liu,Y.(2010). New materials for the flora of Guangxi. *Guihaia.* 30, 448-450&537.(许为斌, 盘波, 梁永延, 朱运喜 & 刘演 (2010) 广西植物区系新资料. 广西植物, 30, 448-450+537.)

Yang,C.H.,Chen,J.Y.,Dai,X.Y.(2011). New Records of Primula in Guizhou. *Guizhou Science.* 29, 32-33&46.(杨成华 & 陈景艳 & 戴晓勇 (2011) 贵州报春花属植物的新分布. 贵州科学, 29, 32-33+46.)

Yang,H.S.,Wang,C.B.,Niu,J.W.,Zhang,S.P.(2013). Vascular Plant Resources and Their Diversity in Liangshui National Nature Reserve Area. *Heilongjiang Agricultural Sciences*.1, 68-70.(杨洪升, 王长宝, 牛校伟 & 张守平 (2013).凉水国家级自然保护区维管植物资源及其多样性. 黑龙江农业科学,1,68-70.)

Yang,Q.Z.(1997).*The distribution of plants in Sichuan.* Guizhou Science and Technology Press.Guiyang.(杨钦周.(1997).四川树木分布.贵州科技出版社,贵阳)

Yao,Y.S.& Li,X.M.(2009). Investigation of Wild Vascular Plant Resources in Qingyuan County, Zhejiang Province. Anhui Agricultural Science Bulletin. 15, 34 & 56.(姚应松 & 李学明 (2009) 浙江庆元县野生维管植物资源调查研究. 安徽农学通报(下半月刊), 15, 34+56.)

Ye.D.(2007). Community characteristics and dynamics of the succession sequence of evergreen broad-leaved forest in Damingshan Nature Reserve. *Guangxi University.* pp 96.(叶铎. (2007) 大明山自然保护区常绿阔叶林演替序列群落特征及动态研究., pp 96. 广西大学.)

Ye,K.,Liu,Q.X.,Deng,M.B.(2010).New records of vascular plants in Jiangsu Province(II). *Journal of Plant Resources and Environment.* 19, 89-90.(叶康 & 刘启新 & 邓懋彬 (2010) 江苏省维管植物分布新记录(二). 植物资源与环境学报, 19, 89-90.)

Ye,K.,Liu,Q.X.,Deng,M.B.,Chu,X.F.(2011). New records of vascular plants in Jiangsu Province(III). *Journal of Plant Resources and Environment.* 20, 95-96.(叶康, 刘启新, 邓懋彬 & 褚晓芳 (2011) 江苏省维管植物分布新记录(三). 植物资源与环境学报, 20, 95-96.)

Ye,K.,Liu,Q.X.,Deng,M.B.,Chu,X.F.(2013). New records of vascular plants in Jiangsu Province(V). *Journal of Plant Resources and Environment.*22, 110-111.(叶康, 刘启新, 邓懋彬 & 褚晓芳 (2013) 江苏省维管植物分布新记录(五). 植物资源与环境学报, 22, 110-111.)

Ye,K.,Liu,Q.X.,Deng,M.B.,Xu,Z.L.(2008). New records of vascular plants in Jiangsu Province(I). *Journal of Plant Resources and Environment.*17,70-72.(叶康, 刘启新, 邓懋彬 & 徐增莱 (2008).江苏省维管植物分布新记录(一). 植物资源与环境学报, 17, 70-72.)

Yi,T.P.(1997).*Bamboo flora of Sichuan.* China Forestry Press.Beijing.(易同培.(1997).四川竹类植物志, 中国林业出版社,北京)

Yu,S.X.,Xu.W.B.,Wu,J.Y.,Yu,L.Y.,Huang,Y.F.(2017). *List of Seed Plants in the Karst Area of Yunnan, Guizhou and Guangxi*. China Environment Press.Beijing.(于胜祥,许为斌,武建勇,余丽莹,黄云峰.(2017). 滇黔桂喀斯特地区种子植物名录. 中国环境出版社.北京.)

Yu,X. & Deng,L.L.(2017). Investigation on Resource Distribution of Ficus Plants in Yunnan Province. *Journal of Hubei University for Nationalities(Natural Science Edition).* 35, 176-181.(余潇 & 邓莉兰 (2017) 云南省榕属植物资源分布研究. 湖北民族学院学报(自然科学版), 35, 176-181.)

Yu,X.H.& Cao,W.(2010). Analysis on the Flora of Vascular Plants in the Fenghuang Mountain Nature Reserve. *Journal of Anhui Agricultural Sciences.* 38, 1527-1530&1534.(于兴华 & 曹伟 (2010) 凤凰山自然保护区维管束植物区系分析. 安徽农业科学, 38, 1527-1530+1534.)

Zang,M. & Huang,L.F.(2010). Floristic Analysis on Vascular Plants of Sanqingshan Mountain in Jiangxi Province. *Subtropical Plant Science.* 39, 55-62.(臧敏 & 黄立发 (2010) 江西三清山维管束植物区系分析. 亚热带植物科学, 39, 55-62.)

Zhang,D.X.(2011).*The plant list of Nanling mountains.* Science Press. Beijing.(张奠湘.(2011).南岭植物名录.科学出版社.北京)

Zhang,F.H.(2010). A FLORISTIC STUDY OF VASCULAR PLANT ON MT.BAOHUA NATIONAL FOREST PARK IN JURONG. *Nanjing Agricultural University*. pp 151.(张帆航. (2010).句容宝华山国家森林公园维管植物区系研究., pp 151. 南京农业大学.)

201.Zhang,F.Y.,Xie,W.Y.,Chen,F.,Liu,B.Q.,Chen,Z.H.(2016).Newcords of vascular plants in Zhejiang Province. *Journal of Zhejiang University(Science Edition).* 43, 497-501.(张芬耀, 谢文远, 陈锋, 刘宝权 & 陈征海 (2016) 浙江维管植物分布新记录. 浙江大学学报(理学版), 43, 497-501.)

Zhang,N.N.,Liu,X.S.,Yang,X.,Guan,W.B.(2007). Analysis on Floristic Composition and Flora in Gurigesitai Nature Reserve, Inner Mongolia, China. *Journal of Shenyang Agricultural University.*3,340-344.(张楠楠, 刘兴双, 杨鑫 & 关文彬 (2007) 内蒙古古日格斯台自然保护区植物区系分析. 沈阳农业大学学报,3,340-344.)

Zhang,S.L.(2007). Study of Plant Diversity and Protection in Saihanwula Nature Reserve, Inner Mongolia.*Beijing Forestry University.* pp 172.(张书理. (2007) 内蒙古赛罕乌拉自然保护区植物多样性及其保护研究. pp 172. 北京林业大学.)

Zhang,S.X..Liu,S.B,Zhang,D.G.(2009). Study on Vegetation Plant Diversity of Xiaoxi National Nature Reserve in Hunan. *Life Science Research.* 13, 122-127.(张世鑫 & 刘世彪 & 张代贵 (2009) 湖南小溪国家级自然保护区植物多样性研究. 生命科学研究, 13, 122-127.)

Zhang,X.Q.(2004). Evaluation of Biodiversity Use Value in Songshan Nature Reserve . *Chinese Academy of Forestry.* pp 70.(张晓秋. (2004) 松山自然保护区生物多样性使用价值评估., pp 70. 中国林业科学研究院.)

Zhang,X.Y.(2010). Study on Vascular Plant Species Diversity in Yesanhe Nature Reserve, Hubei. *Huazhong Agricultural University.* pp 173.(张辛阳. (2012) 湖北野三河自然保护区维管植物物种多样性研究., pp 173. 华中农业大学.)

Zhang,Z.Y.(2012). Study on Plant Diversity in Yanchi Bay National Nature Reserve, Gansu. *Gansu Agricultural University.* pp 103.(张志勇. (2012) 甘肃盐池湾国家级自然保护区植物多样性研究., pp 103. 甘肃农业大学.)

Zhao,H.C. & Feng,B.T.(1996). *Fruit flora of china(Vol. Hawthorn)*. China Forestry Press.Beijing.(赵焕谆,丰宝田. (1996).中国果树志-山楂卷, 中国林业出版社,北京)

Zhao,J.C.,Wang,Z.J.,Li.L.(2005).*Higher flora of Hebei.* Science Press. Beijing.(赵建成,王振杰,李琳. (2005).河北高等植物名录, 科学出版社,北京)

Zhao,L.Q.(2010). New Data of Vascular Plants in Inner Mongolia and Ningxia. *Acta Botanica Boreali-Occidentalia Sinica.* 30, 621-623.(赵利清 (2010) 内蒙古、宁夏维管植物新资料. 西北植物学报, 30, 621-623.)

Zheng,L.X.,Li,P.Y.,Huang,Z.H.(2016). The List of Vascular Plants from Fujian Based on Literature Analysis. *Subtropical Plant Science.* 45, 135-141.(郑丽香 & 李培云 & 黄泽豪 (2016) 基于文献分析的福建维管束植物名录整理. 亚热带植物科学, 45, 135-141.)

Zheng,S.Q.(2013). Plant diversity and evaluation of Daiyunshan National Nature Reserve in Fujian. *Fujian Agriculture and Forestry University.* pp 140.(郑世群. (2013) 福建戴云山国家级自然保护区植物多样性及评价研究., pp 140. 福建农林大学.)

Zhou,D.(2015). Study on Plant Community of Huangsang National Nature Reserve in Hunan. *Central South University of Forestry and Technology.*(周电. (2015) 湖南黄桑国家级自然保护区植物群落研究.中南林业科技大学.)

Zhou,Y.L.(1998). *Flora of Heilongjiang (Vol.4-Vol.11).* Press of Northeast Forestry University. Changchun.(周以良(1998)黑龙江省植物志(4-11卷).东北林业大学出版社.长春)

Zhou,Y.L.(1955). *Xiaoxinganling mountain Woody plants.* China Forestry Press.Beijing.(周以良.(1955).小兴安岭木本植物, 中国林业出版社,北京)

Zi,X.Z.,Zhang,D.C.(2006).*Flora of Dabieshan Mountains.* China Forestry Press.Beijing.(訾兴中,张定成.(2006).大别山植物志. 中国林业出版社,北京)
